# Comparative analyses of dynamic transcriptome profiles highlight key response genes and dominant isoforms for muscle development and growth in chicken

**DOI:** 10.1186/s12711-023-00849-4

**Published:** 2023-10-23

**Authors:** Zhang Wang, Weihua Tian, Dandan Wang, Yulong Guo, Zhimin Cheng, Yanyan Zhang, Xinyan Li, Yihao Zhi, Donghua Li, Zhuanjian Li, Ruirui Jiang, Guoxi Li, Yadong Tian, Xiangtao Kang, Hong Li, Ian C. Dunn, Xiaojun Liu

**Affiliations:** 1 College of Animal Science and Technology, Henan Agricultural University, No. 63, Nongye Road, Zhengzhou, 450002 China; 2 Henan Innovative Engineering Research Center of Poultry Germplasm Resource, Zhengzhou, 450002 China; 3 International Joint Research Laboratory for Poultry Breeding of Henan, Zhengzhou, 450002 China; 4 The Roslin Institute and Royal (Dick) School of Veterinary Studies, University of Edinburgh, Edinburgh, EH25 9RG UK

## Abstract

**Background:**

Modern breeding strategies have resulted in significant differences in muscle mass between indigenous chicken and specialized broiler. However, the molecular regulatory mechanisms that underlie these differences remain elusive. The aim of this study was to identify key genes and regulatory mechanisms underlying differences in breast muscle development between indigenous chicken and specialized broiler.

**Results:**

Two time-series RNA-sequencing profiles of breast muscles were generated from commercial Arbor Acres (AA) broiler (fast-growing) and Chinese indigenous Lushi blue-shelled-egg (LS) chicken (slow-growing) at embryonic days 10, 14, and 18, and post-hatching day 1 and weeks 1, 3, and 5. Principal component analysis of the transcriptome profiles showed that the top four principal components accounted for more than 80% of the total variance in each breed. The developmental axes between the AA and LS chicken overlapped at the embryonic stages but gradually separated at the adult stages. Integrative investigation of differentially-expressed transcripts contained in the top four principal components identified 44 genes that formed a molecular network associated with differences in breast muscle mass between the two breeds. In addition, alternative splicing analysis revealed that genes with multiple isoforms always had one dominant transcript that exhibited a significantly higher expression level than the others. Among the 44 genes, the *TNFRSF6B* gene, a mediator of signal transduction pathways and cell proliferation, harbored two alternative splicing isoforms, *TNFRSF6B-X1* and *TNFRSF6B-X2*. *TNFRSF6B-X1* was the dominant isoform in both breeds before the age of one week. A switching event of the dominant isoform occurred at one week of age, resulting in *TNFRSF6B-X2* being the dominant isoform in AA broiler, whereas *TNFRSF6B-X1* remained the dominant isoform in LS chicken. Gain-of-function assays demonstrated that both isoforms promoted the proliferation of chicken primary myoblasts, but only *TNFRSF6B-X2* augmented the differentiation and intracellular protein content of chicken primary myoblasts.

**Conclusions:**

For the first time, we identified several key genes and dominant isoforms that may be responsible for differences in muscle mass between slow-growing indigenous chicken and fast-growing commercial broiler. These findings provide new insights into the regulatory mechanisms underlying breast muscle development in chicken.

**Supplementary Information:**

The online version contains supplementary material available at 10.1186/s12711-023-00849-4.

## Background

Myogenesis originates from the paraxial mesoderm and refers to embryonic, fetal and adult myogenesis [1]. In chicken, embryonic myoblasts fuse into embryonic myotubes at embryonic day (E) 10–E12, and then fetal myoblasts fuse with embryonic myotubes to form fetal myofibers at E12–E16. At E16–E18, the fetal myofibers continuously fuse to form muscle fibers, and the intramuscular fat is deposited [2]. During the postnatal stages, the muscle fibers are arranged in an orderly pattern, and the number of muscle fibers is fixed. The glycolytic metabolism increases dramatically at 1 day of age (D1) to 1 week of age (W1). At W1–W3, the hypertrophy of the muscle fibers is accompanied by an increase in the number of myonuclei, with the primary source of the new myonuclei being activated and with the addition of satellite cells that are otherwise located on the surface of the muscle fibers [3]. The muscle fibers continue to hypertrophy during W3–W5. Myogenesis is a well-orchestrated process that is controlled by a network of regulatory molecules [4]. Although an increasing number of studies have revealed expression profiles, candidate genes and the regulatory network that are involved in skeletal muscle development [1, 4], the precise regulatory mechanism that underlies myogenesis remains largely elusive.

Comparative transcriptome analysis is a powerful tool for elucidating molecular regulatory mechanisms that underlie complex traits. Many candidate genes and interactive networks have been revealed by analyzing the transcriptomes of individuals that show large phenotypic differences between populations that have the same genetic background, and even among populations with different genetic backgrounds, owing to the continuous development of statistical models and bioinformatic tools [5]. A recent comparison of the transcriptome profiles of the *longissimus* muscle of mutton and traditional local sheep revealed an integrative miRNA-mRNA network that contributed to muscle growth and development [6]. A comparison of the transcriptomic profiles of the *longissimus* muscle between beef and dairy cattle identified differentially-expressed genes that were associated with differences in biological functions between the two breeds, such as muscle development, oxidative phosphorylation, and intramuscular fat (IMF) deposition [7]. In addition, comparative transcriptome studies of skeletal muscle between domestic indigenous and commercial western pigs have elucidated many genes and non-coding RNAs, such as *SATB2* and *XLOC_036765*, that contribute to skeletal muscle diversity via regulation of myoblast migration and proliferation [8, 9]. In chicken, as a result of selective breeding of broilers for meat production in the last ~ 50 years, commercial broilers and indigenous chicken show remarkable phenotypic differences, especially with respect to skeletal muscle mass and IMF content [10]. To explore the genetic basis of skeletal muscle growth and development, and of the IMF deposition in chicken, comparative transcriptome studies on the skeletal muscle of fast-growing commercial broilers and slow-growing indigenous chicken breeds have identified a panel of candidate genes, non-coding RNAs, as well as signaling pathways that are responsible for the phenotypic differences [11–16]. Recently, we studied differences in 3D chromatin architecture between fast-growing broiler and slow-growing indigenous chicken at D1, and discovered the important roles of the *IGF2BP3* and *HMGCR* genes in the regulation of breast muscle development and IMF deposition [17]. Taken together, comparative transcriptomic analyses among various populations can identify key transcripts and regulatory networks that are responsible for phenotypic variation of complex traits.

The Lushi blue-shelled-egg (LS) chicken is a typical dual-purpose indigenous chicken breed with a slow growth rate [18], while the Arbor Acres (AA) chicken is a popular commercial broiler with a fast growth rate. The apparent differences in muscle mass between these two chicken breeds render them ideal models for systematic comparative studies on transcriptional dynamic characteristics during muscle development in chicken.

In the present study, we performed histological and comparative transcriptome analyses of breast muscle between fast-growing broiler (AA) and slow-growing chicken (LS) at seven developmental stages, including three embryonic stages, i.e. at E10, E14 and E18, and four postnatal stages, i.e. at D1, at W1, W3 and W5 [19, 20], and identified the key developmental periods leading to differences in muscle fiber size between these two breeds. Then, we carried out comparative analyses of dynamic transcriptome profiles between the seven time-points and highlighted the key response genes and dominant isoforms involved in muscle development and growth in chicken. This study not only provides new insights into the regulatory mechanism of skeletal muscle development and growth but also provides valuable information for breeding and selection for meat production of indigenous chicken.

## Methods

### Animals and sampling

The LS chickens and fertilized eggs were obtained from the Animal Centre of Henan Agricultural University. The AA broilers and fertilized eggs were obtained from a commercial AA broiler breeding farm. LS and AA birds were raised according to the corresponding feeding standards. Fertilized eggs were incubated in a humidified incubator at 37.5 °C. Seventy healthy embryos were collected at each of the seven sampled developmental stages for sex identification. Fifteen male embryos per breed were harvested at E10, E12, E14, E16, and E18, respectively. Nine male birds per breed were euthanized at D1, W1, W3, and W5, respectively. The left breast muscle tissue of all embryos and birds was quickly collected and frozen in liquid nitrogen, and then stored at − 80 °C for RNA sequencing. The right breast muscle of each embryo and bird was subjected to haematoxylin and eosin (HE) staining. Leg muscle was also collected for DNA isolation and sex identification.

### DNA isolation and sex identification

Genomic DNA was extracted from the leg muscle of the sampled E10, E14, and E18 chicken embryos using the HiPure Tissue DNA Micro Kit (Magen, Guangzhou, China) according to the manufacturer's protocol. DNA integrity and concentration were detected by electrophoresis on a 1.5% agarose gel and Nanodrop 2000 spectrophotometry (Thermo Fisher Scientific, Waltham, USA), respectively. Sex identification was performed by PCR based on the different lengths between the Z and W chromosomes of the sex-linked *chromodomain helicase DNA binding protein 1* (*CHD-1*) gene, as previously described [21]. Genomic DNA was used as template to amplify the *CHD-1* gene to determine the sex of chicken embryos with the forward primer sequence 5′GTTACTGATTCGTCTACGAGA3′, and the reverse primer sequence 5′ATTGAAATGATCCAGTGCTTG3′. PCR was performed in a 10-μL reaction volume containing 1 μL of DNA, 5 μL of 2 × Taq PCR mix (Vazyme, Nanjing, China), 0.5 μL of each forward and reverse primer (10 μM each), and 3 μL of double-distilled water, in a Thermal Cycler Block Instrument (Thermo Fisher Scientific, Waltham, USA). The PCR products were identified by electrophoresis on a 1.5% agarose gel.

### Measurement of the diameter of myofibers

The diameter of myofibers was measured on three samples from AA broiler and LS chicken. Breast muscle tissue was fixed in a 4% paraformaldehyde solution overnight at room temperature. The fixed muscle tissue was embedded in paraffin and cut into 4-μm thick transverse sections. Then, the sections were deparaffinized and hydrated in 100% alcohol for 5 min, followed by 80% alcohol for 5 min. The sections were stained with haematoxylin for 10 min and with eosin for 30 s at room temperature. Three slides from each sample were observed under a light microscope (magnification, × 400). Five fields from each slide were randomly selected to measure the mean diameter of 100 myofibers and 50 myofiber bundles using the DP2-BSW 2.2 software (Olympus, Tokyo, Japan).

### RNA extraction and real time quantitative PCR (RT-qPCR)

Total RNA was extracted from breast muscle tissue and chicken primary myoblasts (CPM) using the TRNzol Universal Reagent Kit (TIANGEN, Beijing, China) according to the manufacturer’s instructions. RNA concentration and integrity were measured using a Nano-Drop 2000 spectrophotometer (Thermo Fisher Scientific, Wilmington, USA). Two μg of each RNA sample with an OD260/280 ratio of 1.9–2.0 and an OD260/230 ratio ≥ 2.0 were reverse-transcribed into cDNA using the HiScript^®^ II 1st Strand cDNA Synthesis Kit (+ gDNA wiper) (Vazyme, Nanjing, China) according to the manufacturer’s instructions. This method includes two steps; in the first step, the gDNA wiper Mix completely removes genomic DNA contamination from the RNA, and in the second step, the HiScript II Enzyme Mix synthesizes cDNA. Fluorescence RT-qPCR was conducted on a Roche LightCycler^®^ 96 Instrument (Roche, Basel, Switzerland) using the AceQ Universal SYBR qPCR Master Mix (Vazyme). Specific qPCR primers were designed and are provided in Additional file 1: Table S1. RT-qPCR was performed in a 10-μL reaction volume containing 1 μL cDNA, 5 μL qPCR Mix, 0.5 μL of each forward and reverse primer (10 μM), and 3 μL of twice-distilled water. The qPCR procedure was performed as follows: 95 °C for 30 s; 35 cycles at 95 °C for 5 s, 59.4 °C for 30 s, and 72 °C for 30 s, followed by 72 °C for 5 min. The housekeeping gene encoding *ACTB* served as internal control for normalizing gene expression. A primer qPCR efficiency ≥ 80% was set for all analyses. All reactions were performed in triplicate. The relative gene expression was quantified using the comparative threshold cycle (2^−ΔΔCT^) method. AA broiler was the calibrator in the analysis of dominant transcript expression between the two breeds, and the control cell group was the calibrator in cell experiments.

### Library preparation and sequencing

Equal amounts of RNA from three embryos or birds at each stage were randomly mixed into one sample, resulting in three biological replicates for RNA sequencing. Three μg of each mixed sample were used to construct the cDNA library after removal of the ribosomal RNA using the Ribominus Eukaryotic kit (Invitrogen, Carlsbad, CA, USA). Subsequently, sequence libraries were generated from the rRNA-depleted RNA using the NEBNext^®^ Ultra™ Directional RNA Library Prep Kit for Illumina^®^ (New England Biolabs, Ipswich, MA, USA), following the manufacturer’s protocol. Briefly, cDNA was synthesized using random hexamer primers and the M-MuLV Reverse Transcriptase (RNase H-). Second-strand cDNA synthesis was performed using DNA polymerase I, and RNase H was used to remove the mRNA. PCR was then performed with the Phusion High-Fidelity DNA polymerase, universal PCR primers, and Index (X) Primer to enrich the cDNA libraries. Finally, cDNA libraries were purified through the AMPure XP system (Beckman Coulter, Fullerton, USA). The effective concentration of the libraries was analyzed using qPCR (library effective concentration > 2 nM). The libraries were sequenced on an Illumina Hiseq 2500 platform (Illumina, San Diego, USA) with a paired-end 150 bp strategy at Novogene Bioinformatics Technology Co., Ltd. (Beijing, China).

### Data filtration and transcriptome assembly

The raw reads were filtered according to the following procedures: (1) adapter sequences were removed, (2) reads with more than 10 unknown bases were eliminated, and (3) low-quality reads with a Qphred score ≤ 20 bases that represent more than 50% of the total read length were removed to obtain clean reads [22]. The base quality and A/T/G/C content of the clean reads were controlled using the fastp software [23]. The percentage of bases with a phred value > 20 (Q20), the percentage of bases with a phred value > 30 (Q30), and the GC content of the clean reads were calculated (see Additional file 2: Table S2). All downstream analyses were based on clean reads.

Clean reads were aligned to the chicken reference genome (GRCg6a) (ftp://ftp.ensembl.org/pub/release-97/fasta/gallus_gallus/dna) using HISAT2 [24] with the − dta option for the transcript assembly using StringTie [25] (v2.1.1), and the output file was stored as.sam files. Samtools was used for the conversion of the.sam file to the.bam file using default parameters [26]. The quality of the transcriptome sequencing was assessed based on sequencing depth and read coverage using the RSeQC 2.6.4 package [27]. Then, the.bam files were imported into StringTie (v2.1.1) with the reference annotation file of chicken (ftp://ftp.ensembl.org/pub/release-97/gtf/gallus_gallus) to assemble and quantify the transcripts.

### Identification of lineage-specific genes in chicken

Conservation of genes expressed in chicken breast muscle was assessed across eight evolutionarily representative species covering six categories, including jawless fish (lamprey), chondrichthyes (elephant shark), osteichthyes (zebrafish), amphibians (tropical clawed toad), reptiles (green lizard), and mammals (prototherian: platypus, and eutherian: human and mouse). For genes expressed in chicken breast muscle, first we produced their corresponding protein sequence files using the TBtools toolkit [28]. Then, orthologous genes in the eight species were identified using the OrthoVenn2 web server with an e-value cutoff of 1e − 5, which is an efficient and interactive graphics tool for genome-wide comparison of orthologous clusters between species [29]. Proteomic files from elephant shark (assembly IMCB_Cmil_1.0) and from the seven other species, including lamprey, zebrafish, tropical clawed toad, green lizard, platypus, mouse, and human, were derived from OrthoVenn2 built-in files.

### Quantification and identification of differentially expressed transcripts

For quantification of gene expression, reads counts of the transcripts in each library were normalized to fragments per kilobase of transcript per million fragments mapped (FPKM) using StringTie (v2.1.1) [25]. The average FPKM of transcripts from three biological repeats represented the expression abundance of transcripts at a certain stage. The genes that exhibited different expression profiles between AA broiler and LS chicken were identified at each time-point. All expressed genes in each sample were used to analyze transcriptional characteristics using the Next-maSigPro [30] (v 3.17) package on the R platform (v 3.8). The effects of breed, developmental stage, and their interaction were taken into account when defining the model [30]. Considering a time-course experiment with T time points and S experimental groups or series, maSigPro uses polynomial regression to model the gene expression value ${y}_{i}$ at condition $i$ and time ${t}_{i}$, and defines S − 1 binary variables (${z}_{S}$) to distinguish between each experimental group and a reference group, using the following model:

1 $${y}_{i}={\upbeta }^{0}+{\upbeta }_{1}{t}_{i}+{\upbeta }_{2}{t}_{i}^{2}+{\upbeta }_{3}{z}_{1i}+{\upbeta }_{4}{t}_{i}{z}_{1i}+{\upbeta }_{5}{t}_{i}^{2}{z}_{1i}+{\epsilon }_{i},$$

where ${\epsilon }_{i}$ is a residual. The *make.design.matrix* parameter [30] was used to set the LS chicken as the reference group and AA broiler as the experimental group. The *p.vector* parameter [30] was used to regress the expression of each gene at each of the seven stages. This parameter also computed *p*-values to identify genes with different expression profiles between the reference group and any other experimental group. By default, maSigPro corrects this *p*-value for multiple comparisons by applying the linear step-up (B-H) false discovery rate (FDR) [30]. The level of FDR control was set to less than 0.05 [30]. The *T.fit* parameter [30] was used to analyze the similarities and differences of the transcriptional characteristics of each gene between the two breeds based on an R-squared threshold of 0.6. The *p.vector* and *T.fit* parameters were estimated based on a general linear model using the least-square approach. The *get.siggenes* parameter [30] was used to extract genes with significantly different expression profiles, which were clustered and visualized by using the *see.genes() command* on “hcluster” [30].

Differentially expressed transcripts (DET) refer to transcripts that are differentially expressed in the breast muscle between AA broiler and LS chicken at a given developmental stage. The reads count matrix of all transcripts was used as an input file to identify DET using edgeR (v3.22.3) [30] on the R platform (v 3.8) based on negative binomial generalized linear models (*glmFit* command), and the expression of transcripts with an absolute value of log2 fold change > 1 and *q*-value < 0.05 at a given developmental stage between two breeds were assigned as DET between the two breeds.

### Gene ontology (GO) enrichment analysis

To identify the genes that are linked to skeletal muscle development, all genes related to growth, development and protein metabolism were detected by GO enrichment analysis using the Blast2GO pipeline with default parameters [32]. GO terms with a *q*-value < 0.05 (Benjamini and Hochberg correction) were considered significant.

### Construction of a developmental axis and comparison of time-series transcriptome

To provide a comprehensive overview of the transcriptomes during breast muscle development, all expressed transcripts in each sample were used to deduce the principal component analysis (PCA)-based axes according to a previous study [33]. The developmental axes for LS chicken and AA broiler were constructed using the R (v 3.8) software, respectively. The input data comprised two sets of transcriptomic profiles containing three biological replicates at seven developmental stages of two breeds. The singular value decomposition (SVD) was employed to construct the developmental axes [33]. For this purpose, the logarithm of the transcripts expression abundance (FPKM) data was arranged into a matrix ${\mathbf{X}}_{\mathrm{sg}}$, with $\mathrm{s}$ columns and $\mathrm{g}$ rows, where $\mathrm{s}$ is the number of samples and $\mathrm{g}$ is the number of quantified transcripts represents. Using SVD, matrix ${\mathbf{X}}_{\mathrm{sg}}$ can be decomposed as:

2 $${\mathbf{X}}_{\mathrm{sg}}=\sum_{{\upalpha }=1}^{\mathrm{s}}{\uplambda }_{{\upalpha }}{\mathbf{w}}_{\mathrm{s}}^{{\upalpha }}{\mathbf{v}}_{\mathrm{g}}^{{\upalpha }},$$

where vectors ${\mathbf{w}}^{{\upalpha }}$ and ${\mathbf{v}}^{{\upalpha }}$ are the singular vectors of matrix ${\mathbf{X}}_{\mathrm{sg}}$. For the SVD decomposition, the $\mathrm{s}\times \mathrm{s}$ matrix can be defined as ${\mathbf{X}}^{\mathrm{T}}\mathbf{X}$, with eigenvalues ${\uplambda }_{{\upalpha }}^{2}$ and eigenvectors ${\mathbf{w}}^{{\upalpha }}$, the g × g matrix as $\mathbf{X}{\mathbf{X}}^{\mathrm{T}}$, with eigenvalues ${\uplambda }_{{\upalpha }}^{2}$ and eigenvectors ${\mathbf{v}}^{{\upalpha }}$, while the other $\mathrm{g}\times \mathrm{s}$ eigenvalues are all equal to zero. The eigenvalues ${\uplambda }_{{\upalpha }}$ are used to define the components of the PCA, the largest eigenvalue, ${\uplambda }_{1}$, representing the first principal component (PC1). Taking PC1 as an example, the eigenvector ${\mathbf{v}}^{1}$ and the gene feature vector ${\mathbf{w}}^{1}$ can be calculated according to Eq. (2). Therefore, the eigenvector ${\mathbf{v}}^{{\upalpha }}$ and the gene feature vector ${\mathbf{w}}^{{\upalpha }}$ of each PC (${\uplambda }_{{\upalpha }}$) can be obtained according to Eq. (2), and the sample eigenvector ${\mathbf{v}}^{{\upalpha }}$ was used to infer a developmental axis. The nonlinear association of ${\mathbf{v}}^{{\upalpha }}$ with each developmental time-point (from E10 to W5) was accessed by fitting LOESS splines using the ggplot2 package [34]. The code required for constructing the developmental axis can be found at https://github.com/zhwang0024/PCA-project. The enriched GO terms of the transcripts that contributed to the top 10% of each axis were used to reflect the functions of the axis. The extent to which a transcript contributed to an axis (contributing value) was determined based on its feature vector: the higher the absolute value of the feature vector (|${\mathbf{w}}^{{\upalpha }}$|), the greater the contribution of the transcript to the axis. An upregulated transcript represented a transcript with a positive feature vector, and conversely, a downregulated transcript represented a transcript with a negative feature vector in the PC axis analysis [31]. The biological functions of the developmental axes were determined by the significantly enriched GO terms of the up-regulated transcripts in each developmental axis.

Because the top four PC explained more than 80% of the variation in both breeds, the biological functions of the developmental axes from the top four PC were analyzed. If the developmental axes were related to skeletal muscle development and did not overlap between AA broiler and LS chicken (at least for one developmental stage), the top 10% contributing transcripts corresponding to these axes were extracted for AA broiler and LS chicken. Transcripts are extracted by the magnitude of their contributing values until the number of transcripts extracted was equal to 10% of the total number of transcripts in each PC, and will be referred to as the top 10% contributing transcripts. The top 10% contributing transcripts that belonged to the PC of AA broilers or LS chicken (also named as AA- or LS-specific top 10% contributing transcripts) were identified.

### Putative protein–protein interactions analysis

All protein sequences used for putative protein–protein interactions (PPI) analysis were obtained from the ENSEMBL database. The PPI analysis was performed using the STRING database (v.10.5) [35]. In brief, the chicken genome was used as reference genome, and all physical subnetworks were displayed according to a confidence score > 0.4 (middle confidence). The PPI networks were constructed using the *cytohubba* plugin in the Cytoscape software (v3.8.2) [36].

### Analysis of alternative splicing events and annotation of dominant isoforms in muscle development

To detect the alternative splicing (AS) events that occurred during transcription, the annotation of quantified transcripts was extracted from the reference annotation file of chicken and inputted into SUPPA2 [37] for alternative splicing analysis. Following Gonzalez-Porta [38], the isoform with a log2 fold change > 1 and a *q*-value < 0.05 relative to any other transcript for a given multi-isoform gene, was assigned as the most dominant isoform. If the FPKM of the other transcripts among the multiple isoforms of the gene was 0, the transcript with the highest expression was the dominant isoform. Dominant isoforms were identified in each of 42 samples and their FPKM of dominant and residual transcripts were normalized and used for comparative analysis of the expression characteristics.

In addition, functional protein motif analysis was conducted using the MEME Suite web server [39] based on the full-length amino acids sequences of transcripts (from the ENSEMBL database) using the following parameters: (1) zero or one occurrence per sequence (zoops); (2) the number of motifs is set to 4; (3) the 0-order model of sequences as background model; and (4) the minimum and maximum widths were set to 6 and 200, respectively.

### In vitro culture and differentiation of chicken primary myoblasts

Isolation of chicken primary myoblasts (CPM) from leg muscle samples of 11-day-embryonic LS chicken was performed according to the standard differential adhesion method [40]. CPM were cultured in DMEM high-glucose medium (Gibco, Grand Island, NY, USA) supplemented with 20% fetal bovine serum (Gibco) and 0.2% penicillin/streptomycin (Gibco) at 37 °C in a 5% CO_2_ incubator. Differentiation of the CPM was induced for two days by culturing the cells in a differentiation medium containing DMEM high-glucose with 2% horse serum instead of fetal bovine serum until they reached 90% confluence.

### Plasmid construction and transfection

To construct overexpression plasmids of the two isoforms of *TNFRSF6B*, the full length of their coding sequences was amplified by PCR with the forward primers including *Kpn*I restriction enzyme sites and reverse primers including *Xba*I restriction enzyme sites (see Additional file 1: Table S1). The PCR was performed in a 20-μL reaction mixture containing 1 μL of cDNA from chicken breast muscle, 10 μL of 2 × Phanta® Flash Master Mix (Vazyme), 1 μL of each of the forward and reverse primers (10 μmol/L), and 7 μL of twice-distilled water. The PCR protocols were as follows: 94 °C for 2 min, 40 cycles at 94 °C for 30 s, 60 °C for 30 s, 72 °C for 30 s, and 72 °C for 5 min. The PCR products were digested with the suitable restriction enzymes, and the purified fragments were ligated into the pcDNA3.1(+) plasmid. After the density of CPM fusion reached 60%, the eukaryotic expression vectors were transfected into the CPM using Lipofectamine™ 3000 Transfection Reagent (Thermo Fisher Scientific, Cleveland, OH, USA) according to the manufacturer’s instructions. The CPM were incubated in a 5% CO_2_ incubator at 37 °C for 36 h to detect proliferation capacity, and for 50 h to detect differentiation capacity.

### Detection of CPM proliferation

The proliferation capacity of CPM was detected by ethynyl-deoxyuridine staining, the CCK-8 cell viability assay and flow cytometry.

For ethynyl-deoxyuridine staining, CPM were treated with 50 μM ethynyl-deoxyuridine (Ribobio, Guangzhou, China) at 36 h post-transfection according to the manufacturer’s protocol and incubated for 2 h at 37 °C in a 5% CO_2_ incubator. The CPM nuclei were stained with 5 μg/mL 4′,6-diamidino-2-phenylindole (DAPI, Ribobio) for 5 min and washed three times with PBS. Cells were observed under a fluorescence microscope (Nikon, Tokyo, Japan).

For the CCK-8 assay, at 12, 24, 36, and 48 h post-transfection, 10 μL of CCK-8 reagent were added to each well (eight wells per group) to detect the absorbance at 450 nm after incubation for 2 h at 37 °C according to the manufacturer’s instructions.

For flow cytometry, after 48 h, the transfected cells were washed twice in a cold phosphate-buffered saline solution and collected in a 1.5 mL centrifuge tube. The cells were then suspended in 500 μL of a propidium iodide (Sigma, MO, USA) solution with 10 μL RNase A (Sigma) at 37 °C for 30 min. The cell cycle phase was detected using a flow cytometer (Becton Dickinson, FACSCa-libur, Franklin, GA, USA), and the proportion of cells in each cell cycle phase was obtained.

### Immunofluorescence

To detect the differentiation of CPM, the immunofluorescence analysis was performed using chicken anti-MYHC as primary antibody (1:50, B103, DSHB, Iowa City, USA) and anti-mouse Cy3-IgG as secondary antibody (ABclonal, Wuhan, China) on CPM as previously described [41]. Cell nuclei were stained with DAPI (Beyotime, Jiangsu, China). Cells were visualized under a fluorescence microscope (Olympus). All analyses were performed in triplicate.

### Determination of cellular protein content

To determine the protein content of the CPM, total DNA and protein were extracted using the Tissue/cell RNA/DNA/protein extraction kit (Bioman, Zhengzhou, China) according to the manufacturer’s instructions. The DNA concentration was determined using a Nano-Drop ND 2000 spectrophotometer (Thermo Fisher). The intracellular DNA content represented the number of cells in each well and was used as an internal control to normalize intracellular protein content. Cellular protein content per well was determined based on the bicinchoninic acid protein (BCA) assay, and the ratio of cellular protein content to cellular DNA concentration per well was used to represent the corrected protein content per well. The average of the corrected protein content (AVE protein content) was calculated in the control cell group, and the following formula was used to calculate the relative protein content of each well:

3 $${\text{Relative protein content per well }} = { 2}^{{ - \, (}{{\text{corrected protein content per well}} - {\text{ AVE protein content}})}} .$$

Each group of CPM contains six biological replicates, and the average value of the relative protein content in a group represents the relative protein content of the group.

### Statistical analyses

Using a completely randomized design, the embryos and birds were randomly grouped and sampled according to the random number table method for all experiments [42]. Each replicate served as the experimental unit for each statistical analysis. For the cell biology experiments, the experimental data were expressed as mean ± standard error of the mean (SE). Statistical significance between two experimental groups was evaluated by a t-test for comparisons using the SAS 9.1.3 software (SAS Institute Inc., NC). Statistical significance is indicated based on the *p*-value: *p-value < 0.05, **p-value < 0.01, ***p-value < 0.001.

## Results

### Comparison of body size parameters and muscle fiber morphometry between AA broiler and LS chicken

**Fig. 1:**
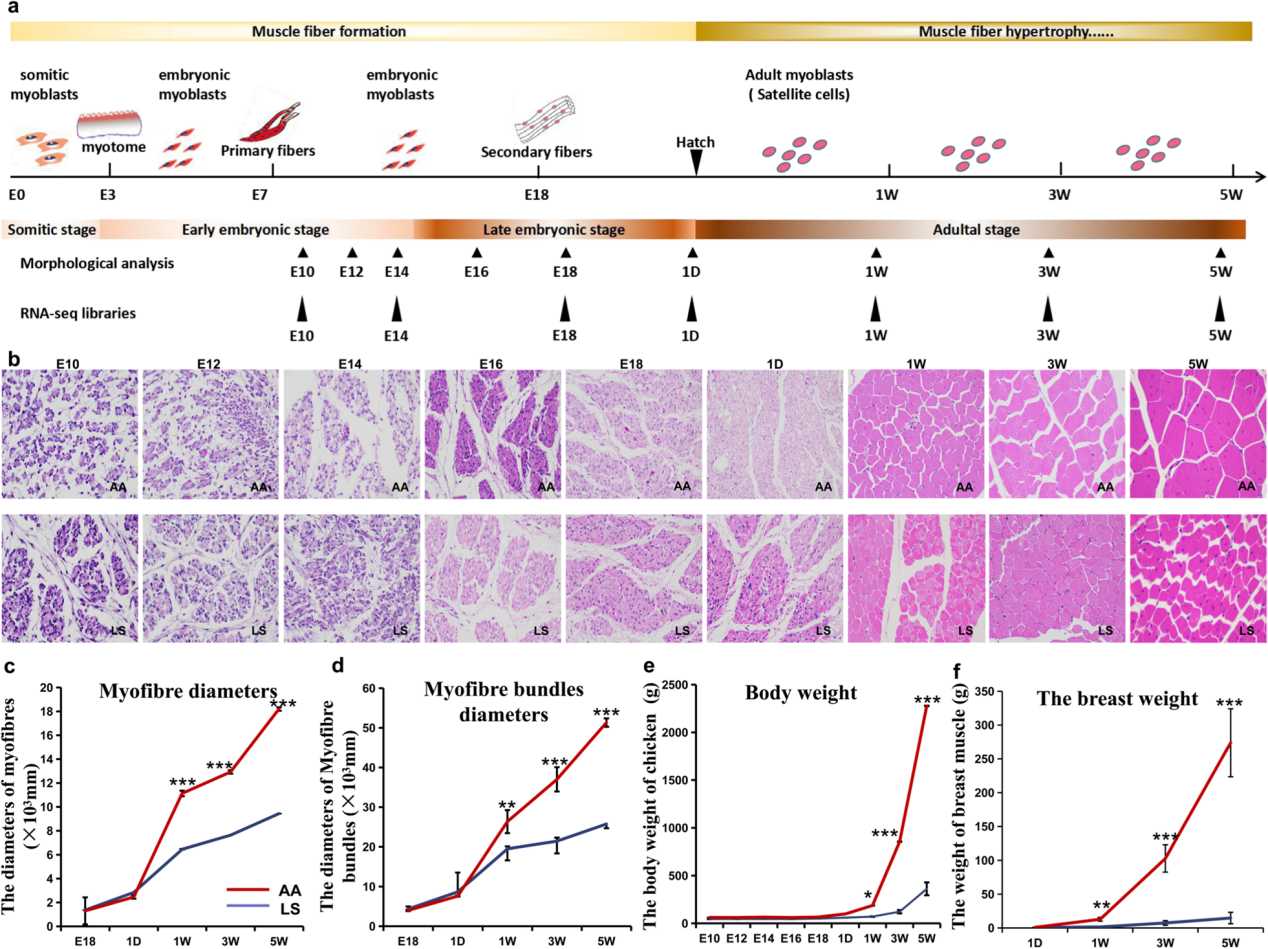
Histomorphological and body size analysis during breast muscle development of AA broiler and LS chicken. **a** Characteristics of breast muscle development from embryonic to adult stage in chicken. **b** Hematoxylin–eosin staining analysis of muscle fiber morphometry during breast muscle development in AA and LS chickens. **c**, **d** Comparative analysis of myofiber diameter and myofiber bundle diameter of the breast muscle between AA broiler and LS chicken. **e**, **f** Comparative analysis of body and breast muscle weights between AA and LS chickens

To evaluate differences in breast muscle development between AA broiler and LS chicken, the characteristics of muscle fiber morphology and body size at the early embryonic stages (E10, E12, E14), late embryonic stages (E16, E18), hatching (D1), and adult stages (W1, W3, and W5) were investigated (Fig. 1a). The results showed that the morphology of the myofibrils varied across the seven developmental stages for both breeds. At E10, the muscle cells migrated directionally in LS chicken, and the initial muscle fiber bundle appeared at E12, whereas in AA broiler they did not form before E14 (Fig. 1b). Significant differences in myofiber diameter and myofiber bundle diameter between AA broiler and LS chicken were first observed after hatching at W1 and continued until W5, with significantly larger myofiber and myofiber bundle diameters in AA broiler than in LS chicken (Fig. 1c, d). Moreover, several body size parameters, including body weight and breast muscle weight, were significantly greater in AA broiler than in LS chicken (Fig. 1e, f) and (see Additional file 3: Fig. S1). These results imply that LS chicken showed earlier myogenesis than AA chickens during the embryonic period. However, after hatching, myofiber hypertrophy was more pronounced in AA broiler than in LS chicken. These significant differences in muscle fiber and body size characteristics between AA broiler and LS chicken appeared at W1 and rapidly increased after hatching at W3.

### Overview of the transcriptomes during muscle development

To understand the molecular regulatory mechanism underlying skeletal muscle development, we profiled the transcriptome of breast muscle across seven developmental time-points in AA and LS chickens. After removing adapter sequences, reads with more than 10% unknown bases, and low-quality reads, 3875.2 × 10^6^ clean reads of the 4034.4 × 10^6^ raw reads were available. On average, clean reads accounted for up to 96.05% of the raw reads (see Additional file 2: Table S2). In total, 16,878 genes with 28,445 transcripts were identified in the 42 samples, including 13,189 genes with expression levels higher than 1 in at least one sample (see Additional file 4: Table S3). These results indicate that the sequencing quality was highly reliable.

**Fig. 2:**
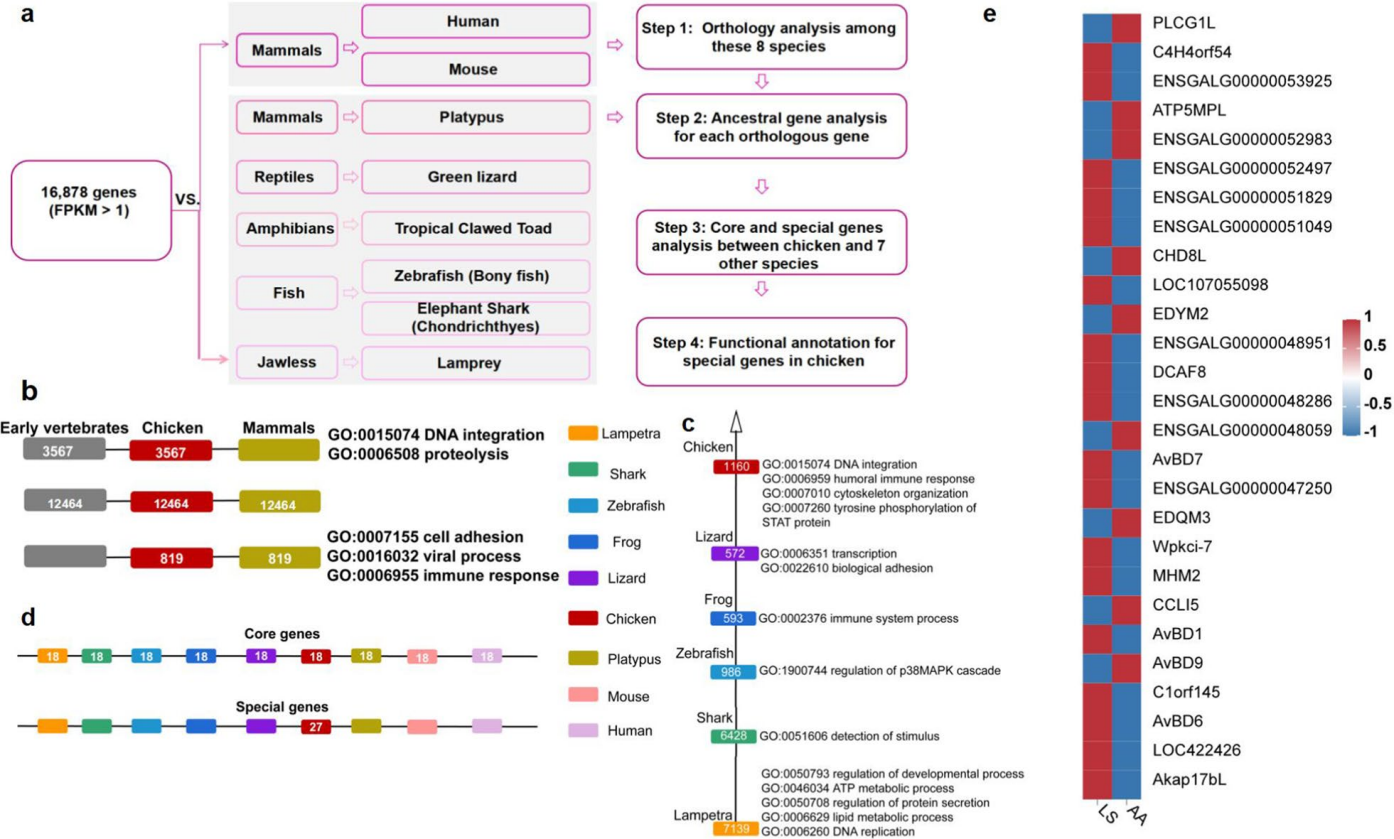
Lineage-specific expansion/contraction of protein-coding gene families. **a** Flow chart of expansion/contraction analysis of protein-coding gene families in the chicken breast muscle. The deeper purple border indicates a later emergence time of the species. **b** Distribution and functional enrichment analysis of genes expressed in chicken breast muscle in various vertebrates (**c**) and species that predate the appearance of chicken. **d** Distribution of the number of genes present in all studied species (upper and core genes) and that of genes present only in chicken (lower, special genes). **e** Heatmap showing the total expression of lineage-specific genes between AA broiler and LS chicken

Conservation of genes expressed in chicken breast muscle across eight evolutionarily representative species identified 13,639 orthologous genes from 16,878 genes (12,826 gene families), of which 12,464 genes were present in all nine species, 3567 genes were not found in mammals, and 819 orthologous genes were retained in chicken and mammals (Fig. 2b). Furthermore, 15,718 (93.1%) of the genes originated during early chicken evolution, since these genes arose during the very early stage of the vertebrate evolution predating the divergence of the bird lineages; and 13,567 genes originated early in the teleost evolution, which were enriched for DNA replication, lipid metabolic process, ATP metabolic processes, detection of stimulus, regulation of the p38MAPK cascade, transcription, and biological adhesion (Fig. 2c). In addition, 27 chicken lineage-specific genes related to immunity were detected in chicken, comprising 17 novel genes and 10 annotated genes (Fig. 2d). These annotated genes, namely, *CCLI5*, *AKAP17BL*, *MHM2*, *EDQM3*, *CHD8L*, *PLCG1L,* and *DCAF8* (see Additional file 5: Table S4), included three members of the *AvBD* gene family. Comparison of the global expression of the lineage-specific genes in the breast muscle between the two chicken breeds revealed 18 upregulated genes and nine downregulated genes in LS chicken compared to AA broiler (Fig. 2e). These results suggest that the majority of the genes discovered in the breast muscle transcriptomes of the AA and LS chickens were orthologous among vertebrates, but some immune-related genes were only found in the chicken lineage and exhibited inconsistent expression patterns between AA and LS chickens.

### Transcriptional characteristics of skeletal muscle at different developmental stages

**Fig. 3:**
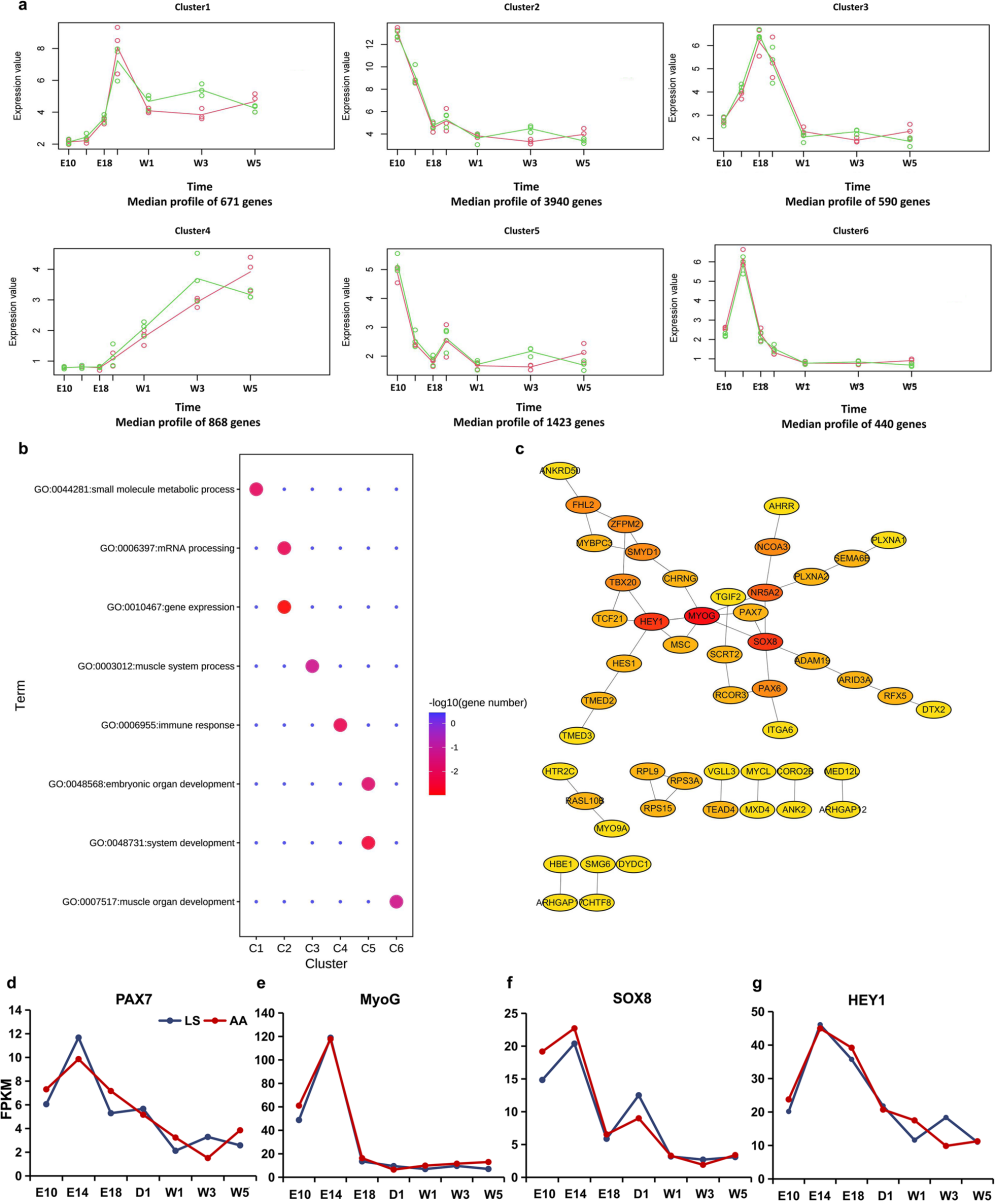
Overview of transcriptional profiles during breast muscle development. **a** Temporal expression profiles of transcripts in the breast muscle between AA and LS chickens. **b** GO enrichment analysis of transcripts in each cluster. (**c**) PPI analysis of genes involved in myogenesis. **d–g** Expression patterns of the *PAX7*, *MYOG*, *SOX8*, and *HEY1* genes in breast muscle at different developmental stages of AA broiler and LS chicken

To explore the characteristics of the temporal expression profiles across seven developmental stages, the time-series profiles of 28,445 genes expressed in breast muscle from the two breeds were classified using the hcluster algorithm. The 10,877 transcripts clustered into nine characteristic transcriptional patterns with high confidence intervals (FDR < 0.05) (see Additional file 6: Table S5). Because the genes (based on the corresponding transcripts) in clusters 3 and 6 were functionally enriched for skeletal muscle development according to GO annotations, the features of these genes, including 21 transcription factor (TF) genes, were analyzed in detail (Fig. 3a, b). Ten of the 21 TF genes were necessary for myogenesis, reflecting the high commonality of myogenic processes in the two breeds. Putative PPI analysis of the TF revealed that the *MYOG*, *HEY1* and *SOX8* genes, which are critical for formation of skeletal muscle and demonstrated similar expression patterns in both breeds across the seven developmental stages, had the strongest network connectivity (Fig. 3c–g). These results indicate that the genes responsible for muscle growth in chicken are expressed in a temporally similar manner in both breeds.

### Comparative transcriptome analyses between AA broiler and LS chicken during muscle development

**Fig. 4:**
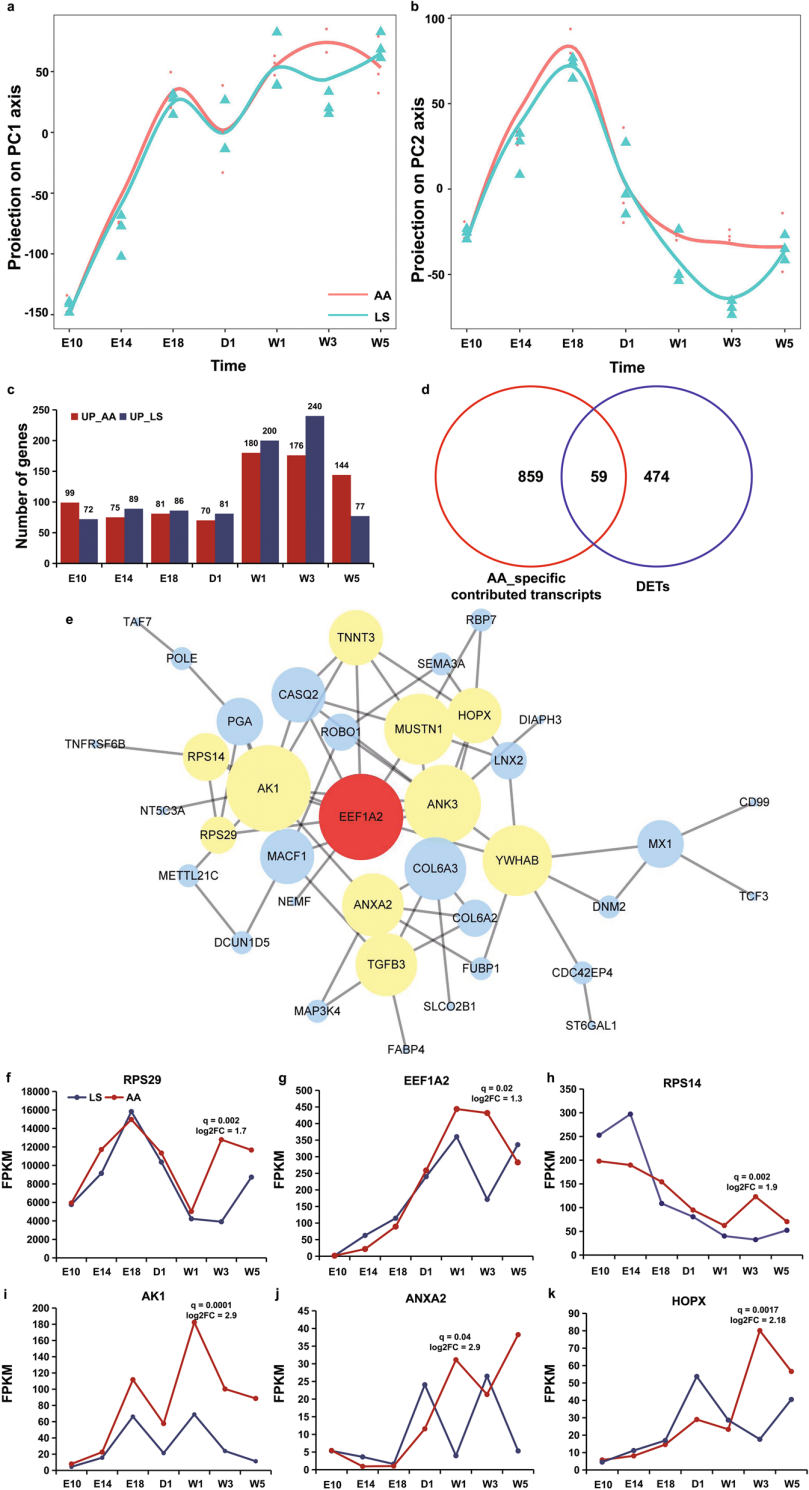
Comparative analysis of gene expression in breast muscles at different development stages between the AA and LS breeds. **a**, **b** Developmental axes from the top two principal components. The red curve represents the developmental axis of AA broiler, and the blue curve represents the developmental axis of LS chicken. The X-axis represents the seven developmental stages, and the Y-axis represents the eigenvector. **c** Number of DET detected in the breast muscle between AA and LS chickens. **d** Venn analysis of DET and AA-specific transcripts. **e** PPI analysis of the genes identified by Venn analysis. **f–i**, **g**, **k** Expression patterns of *RPS29*, *EEF1A2*, *RPS14*, *AK1*, *ANXA2,* and *HOPX* in breast muscle at different developmental stages of AA broiler and LS chicken

To accurately identify the genes that positively affect morphometric differences in breast muscle between AA broiler and LS chicken, we first analyzed the developmental axis of the two breeds by fitting the eigenvector curves on the time series. The top four principal components (PC1 to PC4) explained more than 80% of the variation in both breeds (see Additional file 7: Fig. S2). Comparative analysis clearly showed that the developmental axes of the two breeds overlapped at embryonic stages E10 to E18 but gradually separated through the adult stages, with the greatest separation between the two breeds observed in the top four PC between 1 and 3 weeks of age (Fig. 4a, b) and (see Additional file 8: Fig. S3a, b). Considering that the eigenvector of a physiological period is related to the coefficient of variation (also known as the contribution value) of transcripts in that period, we identified the top 10% contributing transcripts for each principal component (see Additional file 8: Fig. S3c, d; Additional file 9: Table S6). In total, 5353 top 10% contributing transcripts from 4349 genes in the top four PC for LS chicken were significantly enriched for myogenesis-related GO terms, and 4367 top 10% contributing transcripts from 3674 genes were significantly enriched for skeletal muscle development- related GO terms in the top three PC for AA broiler (see Additional file 8: Fig. S3e; Additional file 9: Table S6). Nine-hundred and eighteen transcripts from 861 genes were in the top three PC of AA broiler but not in the top four PC of LS chicken, while 1903 transcripts were in the top four PC of LS chicken, but not in the top three PC of AA broiler (see Additional file 8: Fig. S3f; Additional file 9: Table S6). These results suggest that the former 918 transcripts may be the main contributors to the differences in skeletal muscle development during the postnatal physiological period between AA broiler and LS chicken.

Differentially expressed transcripts between the two chicken breeds were identified for each developmental stage. More changes in transcript expression from one time point to the next were found at the post-hatching stages than at the embryonic stages (Fig. 4c). Differentially expressed transcripts between AA and LS chickens at the embryonic stages were significantly enriched for neuron development or hormone secretion-associated gene terms (see Additional file 10: Fig. S4a, c; Additional File 11: Table S7). At the post-hatching stage, the DET included 533 and 581 upregulated and downregulated transcripts in AA broiler, corresponding to 480 and 529 genes, respectively (see Additional file 10: Fig. S4b; Additional file 11: Table S7). GO enrichment analysis showed that the upregulated DET in AA broiler were significantly enriched for the following terms: regulation of developmental growth genes on day 1 after hatching, cellular amino acid biosynthetic process genes at one week of age, and muscle development genes after hatching at weeks 3 and 5 (see Additional file 10: Fig. S4d). These findings are consistent with the significant morphometric differences in breast muscle between these two breeds. Fifty-nine transcripts from 58 genes were identified based on a combination of the above 918 transcripts and the 533 upregulated transcripts in AA broiler (Fig. 4d) and (see Additional file 12: Table S8). Among these 58 genes, based on the muscle development associated gene identification analysis, we found that 44 genes, including *RPS29*, *EEF1A2*, *RPS14, AK1*, *ANXA2* and *HOPX*, formed a molecular network that is involved in muscle development and protein synthesis (Fig. 4e–k). Therefore, these 44 genes act as core genes responsible for muscle development and might underlie differences in muscle content between fast-growing AA broiler and slow-growing LS chicken.

### Dynamic alternative splicing events and changes in dominant isoforms during skeletal muscle development

A subset of 17,781 transcripts from 6215 multi-isoform genes was used to analyze AS events during breast muscle development (see Additional file 13: Table S9). Alternative splicing events were more frequent at the embryonic stages than at the post-hatching stages, with the smallest number of AS events observed at week 1 after hatching (see Additional file 14: Fig. S5a). Using gene structure annotation for the isoforms of each gene, seven main types of AS events in chicken breast muscle were investigated. Exon skipping was the most frequent AS event, with approximately 28% of all AS events, and the mutually exclusive exons had the lowest rate (3%) (see Additional file 14: Fig. S5b, c).

**Fig. 5:**
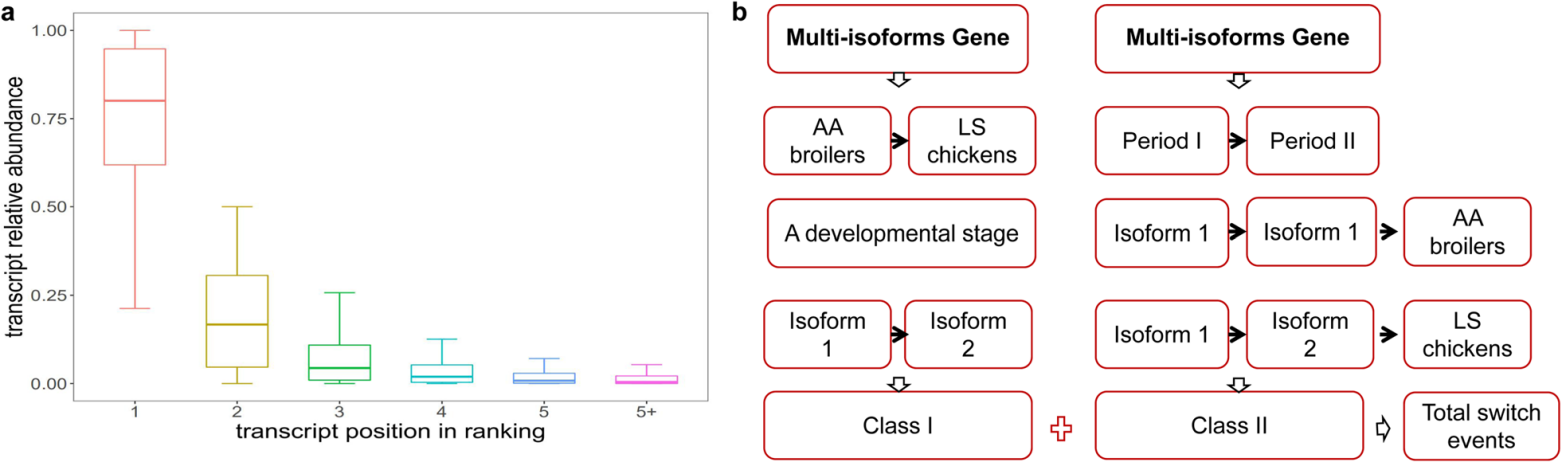
Identification of dominant transcripts in breast muscle between the AA and LS breeds at different developmental stages. **a** Relative expression abundance of the subset of transcripts at each position in the ranking of dominant transcripts. For each gene, transcripts were ranked based on their relative abundance. **b** Flow chart of the identification of different dominant transcripts between AA and LS chickens

To further investigate the functional mechanism of the AS events in myogenesis, the dominant isoform for each multi-isoform gene expressed in the breast muscle of AA broiler and LS chicken across the seven developmental stages was analyzed. The results indicated that one transcript, termed the dominant isoform, was often expressed at a significantly higher level (log2 fold change > 1 and *q*-value < 0.05) than any other transcript within a given multi-isoform gene at each developmental stage. The dominant isoforms were produced by exon skipping, and the production of dominant isoforms was independent of the number of transcripts (Fig. 5a) and (see Additional file 15: Fig. S6a). In LS chicken, 6240 transcripts (6151 genes) were identified as the dominant isoform for at least one developmental stage, whereas 6248 dominant isoforms (6156 genes) were identified in AA broiler (see Additional file 16: Table S10). Intriguingly, 88 genes (176 isoforms) in LS chicken and 84 genes (174 isoforms) in AA broiler had different dominant isoforms across the seven developmental stages (see Additional file 13: Table S9). At a certain stage, there was only one dominant transcript for each multi-isoform gene. For example, the dominant transcript of the *SOAT1* gene at post-hatching day 1 in LS chicken was ENSGALT00000006691, while it was ENSGALT00000060114 at post-hatching week 1 (see Additional file 15: Fig. S6b). These results demonstrate that the dominant transcripts of multi-isoform genes can switch during muscle development in chicken.

Considering that the different dominant transcript switching events between the two breeds might be related to the differences in phenotypic characteristics of their skeletal muscle, we divided them into two classes. The first class (class I) of switch events was characterized by genes with dominant isoforms that differed between the two breeds during the same developmental stage. One hundred and five dominant isoforms from 50 multi-isoform genes demonstrated class-I switch events. The second class (class II) of switch events was characterized by genes with dominant isoforms that differed between AA broiler and LS chicken for any two of the seven developmental stages. In total, 101 dominant transcripts from 45 genes showed class II switch events (Fig. 5b) and (see Additional file 17: Table S11). Collectively, 85 genes underwent dominant transcript switch events between the two breeds (see Additional file 17: Table S11). These findings show that LS and AA chickens have different dominant isoforms for certain genes at specific developmental stages.

### Switching of the dominant isoform of the *TNFRSF6B* gene affects skeletal muscle development

**Fig. 6:**
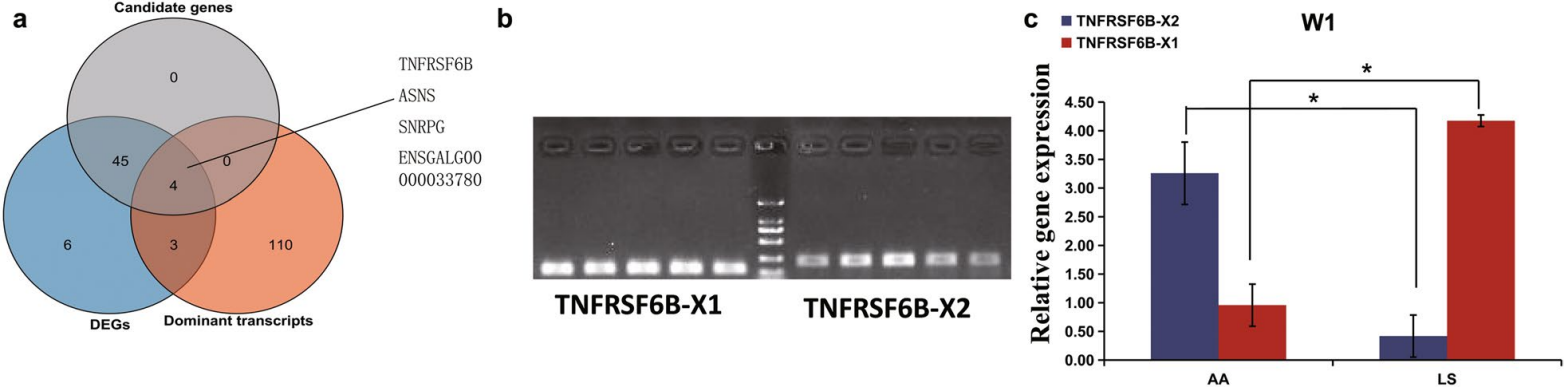
Dominant transcripts of *TNFRSF6B* exhibited a switched event between the AA and LS breeds. **a** Venn diagram of DET, switched dominant transcripts and 44 candidate genes in breast muscle between the AA and LS breeds. The samples amplified by PCR were breast muscles of LS chicken at day 1. The five lanes at the left of the DNA marker were the PCR amplification results of *TNFRSF6B*-X1, and at the right side those of *TNFRSF6B*-X2. **b** PCR amplification of the two isoforms of *TNFRSF6B*. **c** qPCR detection of the expression of the two isoforms at W1 in AA and LS chickens

To further investigate the effect of dominant transcript switching events on morphometric differences in breast muscle between the two breeds, we carried out an integrative analysis of the DET, the genes undergoing dominant transcript switching, and the aforementioned 44 candidate genes, and we identified four genes which exhibited a different dominant transcript switching event during muscle development between AA broiler and LS chicken (Fig. 6a). Among these, the *TNFRSF6B* gene showed two isoforms, *TNFRSF6B*-X1 and *TNFRSF6B*-X2, with the different 5’ untranslated region (UTR) sequences and exon 1 sequences, leading to the 135 bp longer coding sequence of *TNFRSF6B*-X1 compared to that of *TNFRSF6B*-X2 (see Additional file 18: Fig. S7a). Then, we designed the specific primers for the 5’UTR sequence of *TNFRSF6B-X1* and exon 1 sequence of *TNFRSF6B-X2*, respectively, to distinguish between*TNFRSF6B-X1* and *TNFRSF6B-X2* (Fig. 6b). The expression levels of *TNFRSF6B-X1* and *TNFRSF6B-X2* were similar in both AA and LS chickens at day 1 after hatching (see Additional file 18: Fig. S7b, c). However, *TNFRSF6B-X2* became the dominant transcript in AA broiler at week 1 after hatching, whereas *TNFRSF6B-X1* remained the dominant transcript in LS chicken (Fig. 6c).

**Fig. 7:**
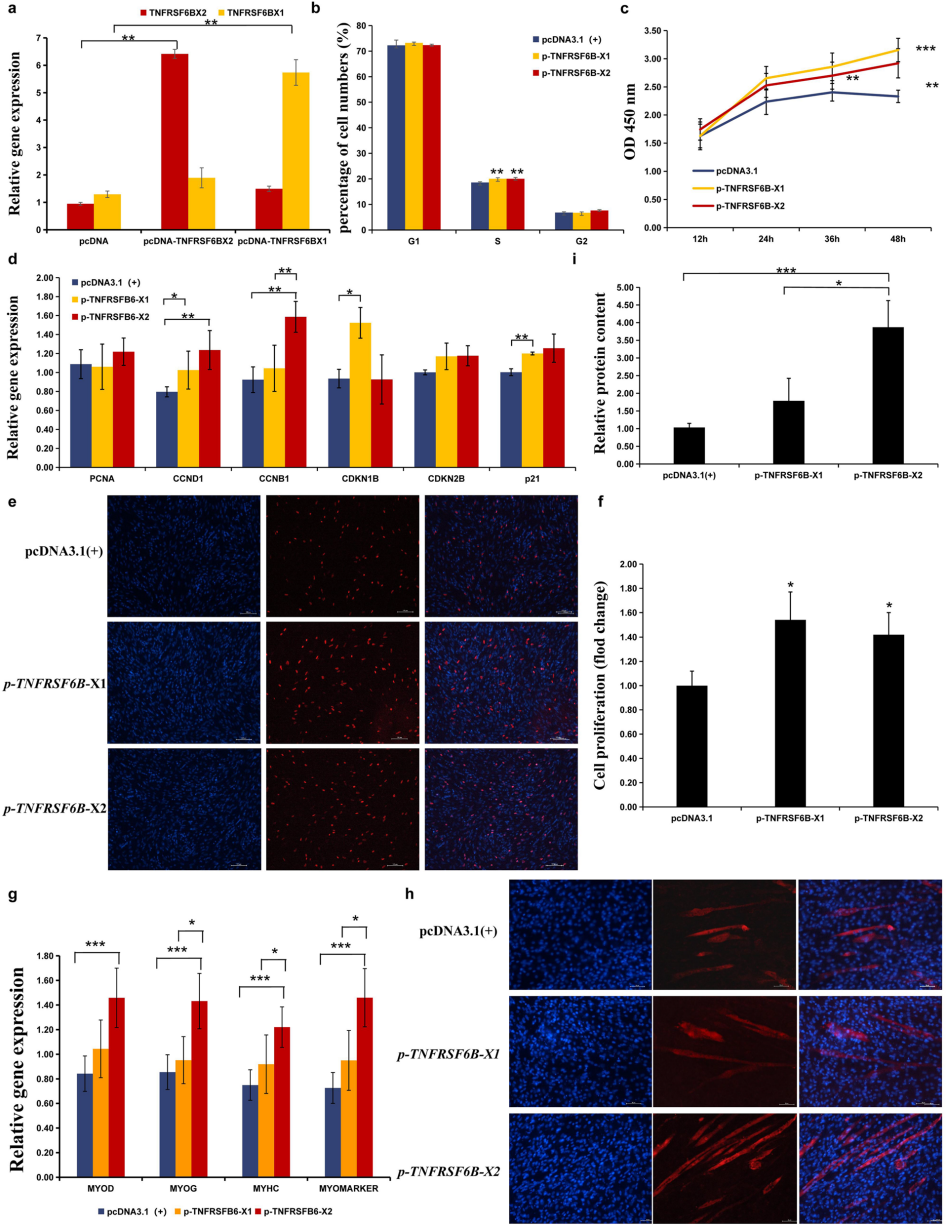
Switching of dominant transcripts of *TNFRSF6B* affects skeletal muscle development between the AA and LS breeds. **a** qPCR detection of the overexpression of the two isoforms in chicken primary myoblasts (CPM) for 24 h. Effects of the overexpression of the two isoforms on the proliferation of CPMs as determined by flow cytometry (**b**) and CCK-8 assay (**c**, **d**) Relative expression of genes involved in cell proliferation after overexpression of the two isoforms in CPM. **e** Detection of novel cells after overexpression of the two isoforms via ethynyl-deoxyuridine assay, respectively. **f** The number of novel cells after overexpression of the two isoforms, respectively. **g** Relative expression of genes involved in cell differentiation after overexpression of the two isoforms in CPM. **h** Immunofluorescence detection of differentiated CPM. Red represents differentiated myotubes, and blue represents cell nuclei. **i** Relative protein content after overexpression of the two isoforms in the CPM. * Represents *p* < 0.05, ** represents *p* < 0.01, *** represents *p* < 0.001

To further understand the biological functions of these two *TNFRSF6B* isoforms during myofiber development, an in vitro experiment in CPM was performed. Overexpression of the *TNFRSF6B-X1* and *TNFRSF6B-X2* isoforms significantly increased the proportion of S-phase myoblasts and the number of living myoblasts compared to those in the control cell group (*p*-value < 0.05) (Fig. 7a–c). The number of ethynyl-deoxyuridine-positive CPM also increased, accompanied by the upregulation of several cell cycle-related genes, including *PCNA*, *CCND1*, *CCNB2*, *CDKN1B*, *CDKN2B*, and *P21* (Fig. 7d–f). The effects of *TNFRSF6B-X1* and *TNFRSF6B-X2* on CPM proliferation were not significantly different from each other (*p*-value > 0.05). However, RT-qPCR results showed that overexpression of the *TNFRSF6B-X2* isoform significantly increased expression of the marker genes responsible for myoblast differentiation, including *MYOD*, *MYOG*, *MYHC,* and *MYOMARKER*, compared to the control cell group, but overexpression of the *TNFRSF6B-X1* isoform had no significant effect on the expression of these marker genes (Fig. 7g). Immunofluorescence assays showed that overexpression of *TNFRSF6B-X2*, significantly increased differentiation and fusion of CPM compared to the control cell group but *TNFRSF6B-X1* overexpression did not (Fig. 7h). In addition, the intracellular protein content in the CPM that overexpress the *TNFRSF6B*-X2 isoform was significantly greater than that in the control CPM and in those that overexpressed the *TNFRSF6B*-X1 isoform. However, the intracellular protein content was not affected by *TNFRSF6B*-X1 isoform overexpression (Fig. 7i). These results suggest that, although the two isoforms of *TNFRSF6B* promote myoblast proliferation, only the *TNFRSF6B*-X2 isoform promotes myoblast differentiation and fusion, suggesting that the dominant isoform *TNFRSF6B*-X2 accelerates myogenesis in AA broiler from W1 onward, leading to the subsequent differences in breast muscle fiber morphology between AA broiler and LS chickens.

## Discussion

The body weight of fast-growing birds, especially breast muscle weight, is at least three-fold higher than that of slow-growing birds, although embryonic myogenesis in slow-growing birds begins earlier [18]. Throughout myogenesis, orientation of muscle fibers and subsequent formation of a single muscle bundle are necessary steps for muscle fiber development [43]. In this study, histomorphological analysis of breast muscle revealed that myogenesis starts earlier during early embryonic stages in the slow-growing LS chicken than in the fast-growing AA broiler, in agreement with previous studies in birds [17] and mammals [7]. Nonetheless, after hatching, a striking increase in weight gain, myofiber diameter, and myofiber bundle diameter was observed in the fast-growing AA broiler compared to the slow-growing LS chicken. This increase begins at W1 and rapidly expands at W3. Therefore, it was speculated that certain periods after hatching, such as post-hatching W1 and 3 were the key stages leading to the difference in muscle mass between slow-growing LS chicken and fast-growing AA broiler.

However, rapid growth is associated with several detrimental consequences. In addition to heart and musculoskeletal problems, which are direct consequences of additional weight, the immune response is also thought to be altered in modern broiler [8], and a previous study inferred that improving growth performance occurs at the expense of immune functions [44]. Our study showed that genes expressed in skeletal muscle that are specific to a chicken lineage are the result of the expansion of the family of genes involved in the immune system, and most lineage-specific genes related to immunity were upregulated in the breast muscle of LS chicken compared to that of AA broiler, suggesting that the rapid accumulation of breast muscle mass in AA broiler may be related to changes in immune functions. However, the detailed mechanisms still require further study.

Muscles expand by an increase in the number of myofibers (hyperplasia), which mainly occurs during the embryonic period, and an increase in the size of myofibers (hypertrophy), which is accompanied by enhanced protein synthesis and occurs primarily after hatching [45]. Using the hcluster approach, genes related to energy metabolism, protein metabolism, RNA processes, and skeletal muscle development were found to have similar expression profiles in both breeds. Functionally similar genes tend to exhibit co-expression patterns [46]. In this study, the genes related to skeletal muscle development, including 10 TF genes essential for myogenesis, such as *MYOG, HEY1,* and *SOX8* [47, 48], had similar expression patterns in the breast muscle of both breeds. However, this approach did not identify muscle-related genes which exhibited different transcriptional characteristics between the two breeds.

PCA is often used to assess the degree of differences between groups in transcriptome studies, which are mainly related to the top contributing transcripts for each PC. The greater is the difference in gene expression levels between two groups, the greater is the separation between the groups based on PCA. Previous PCA of developmental axes enabled the discovery of differences in time-series transcriptomes between different treated groups [33]. In this study, developmental axis was used to identify key periods and key genes that contribute to differences in muscle development between the two breeds. The period from 1 to 3 weeks old was considered as the key developmental stage that contributes to differences in breast muscle development between LS chicken and AA broiler, as demonstrated by the analysis of the developmental axis based on molecular expression characteristics. These findings are consistent with the morphological differences observed at these stages between the two breeds.

Using gene sets generated from the combined study of developmental axes and DET, it was possible to identify candidate genes that may be responsible for the differences in muscle mass between the two chicken breeds. Putative PPI analysis revealed several upregulated genes in AA broiler, that play pivotal roles in regulating muscle growth and hypertrophy as strong stimulators of myoblast and satellite cell proliferation and differentiation, such as *TNNT3* [49], *MUSTN1* [50], *HOXP* [51], *ANXA2* [52], and *AK1* [53]. In addition, the *EEF1A2* gene, which encodes a protein involved in the elongation step of protein synthesis [54], as well as the *RPS14* and *RPS29* genes, which encode ribosomal proteins [55], can respond to the mTOR signaling pathway and subsequently regulate the accumulation of proteins in skeletal muscle, thereby leading to myofiber hypertrophy [56, 57]. Therefore, these genes could be considered candidates for improving muscle mass in indigenous chicken.

Alternative splicing is a post-transcriptional process that generates multiple transcripts from a single precursor mRNA molecule and sequential protein variants, thereby playing a dynamic regulatory role in many biological processes [58]. Although many studies of RNA-seq data have revealed that AS events may also participate in the regulation of myogenesis, the general understanding of differential isoform usage remains poor [58–61]. Trapnell et al. [62] who investigated the link between differential isoform usage and myogenesis found that many genes underwent switching between major and minor isoforms based on the frequency of their occurrence during the myogenic differentiation of mouse skeletal muscle C2C12 cells. To date, no studies have systematically compared the differences in isoform switched events between breeds. Here, we comprehensively analyzed AS events during chicken muscle development and identified different dominant isoforms switching events between the two chicken breeds. The results showed that the characteristics of AS events during skeletal muscle development in chicken are very similar to those in mammals, with the largest proportion consisting of skipped exons and the smallest proportion consisting of mutually exclusive exons [63]. Another important feature of dominant isoforms is that only one dominant isoform was observed for most genes in breast muscle at each developmental stage in both AA and LS chickens, which is consistent with the results of [64] on mammals. In this study, some genes had multiple dominant isoforms at different developmental stages, showing that the dominant isoforms of these genes were switched during skeletal muscle development. AS often leads to the gain or loss of a protein domain, of an open reading frame, or of signal peptides, suggesting that AS events may alter the function of genes [38, 65–69]. To further investigate this, we defined the switched dominant isoform in the breast muscle between the two chicken breeds at each developmental stage. Many of these switching events have been previously described in humans and affect the developmental process of skeletal muscle, cell communication, transcriptional regulator activity and regulation of metabolism [70]. These results imply that the switched dominant isoforms were possibly associated with differential muscle mass between the two chicken breeds evaluated here.

TNFRSF6B, a tumor necrosis factor receptor superfamily member 6b, has been associated with osteoclastic activity, inhibition of apoptosis, and modulation of T cell activation and differentiation [71]. The present study found that the *TNFRSF6B* gene underwent a switch of the dominant isoform between the two breeds at W1. At W1, *TNFRSF6B-X2* was the dominant isoform in AA broiler, and *TNFRSF6B-X1* the dominant isoform in LS chicken. These two isoforms of *TNFRSF6B* were overexpressed in chicken myoblasts, and both transcripts were found to promote myoblast proliferation, which may be related to their ability to reduce apoptosis. Myoblast differentiation is a key process that ensures the maturation of muscle fibers. Overexpression of the *TNFRSF6B-X2* isoform, which was observed to be the dominant isoform in AA broiler, significantly increased myoblast differentiation in both breeds. However, overexpression of the *TNFRSF6B-X1* isoform, which was the dominant isoform in LS chicken, failed to affect myoblast differentiation. Muscle fiber development after hatching relies mainly on hypertrophy, which is induced by an increase in protein synthesis and characterized by an increase in muscle fiber size. Although marker genes for hypertrophy have not been well examined [14], intracellular protein content can be measured and corrected based on the DNA concentration per well. In this study, overexpression of *TNFRSF6B-X2* significantly increased total intracellular protein in adult myoblasts but overexpression of *TNFRSF6B-X1* had no significant effect. These results suggest that the switching events of the dominant isoform of some genes play an important role in breast muscle development and that the *TNFRSF6B*-X2 isoform may contribute to the differences in breast muscle mass between AA broiler and LS chicken.

## Conclusions

In this study, we identified a set of genes and dominant transcripts that are involved in skeletal muscle development and growth in chicken. These findings not only provide insights into the regulatory mechanism of skeletal muscle development and growth but also provide valuable resources for genomic selection on muscle mass via molecular breeding in the chicken industry.

### Supplementary Information

**Additional file 1: Table S1.** Information on the primer sequences used in this study.

**Additional file 2: Table S2.** Summary of sequencing quality: raw reads, clean reads, mapping rate, Q20 and Q30 in this study.

**Additional file 3: Figure S1.** Comparison of body size phenotypes: (**a**) TC: tibial circumference; (**b**) BSL: body slanting length; (**c**) CW: breast width; (**d**) CD: breast depth; (**e**) KL: Keel length; (**f**) PW: pelvis width; (**g**) TL: tibial length.

**Additional file 4: Table S3.** List of all qualitative transcripts and the genes with expression levels higher than 1.

**Additional file 5: Table S4.** Evolutionary analysis. The numbers used to name the sublists correspond to the number of different types of the orthologous genes in Fig. 2.

**Additional file 6: Table S5.** Transcriptional characteristics of skeletal muscles between the two breeds for seven developmental stages. The 10,877 transcripts from this transcriptome were clustered into nine characteristic transcriptional patterns. If the transcript was identified an transcriptional factor (TF), this transcript was marked “True”. Each transcript was grouped into a cluster, and marked 1 to 9, respectively.

**Additional file 7: Figure S2.** Top 4 principal components (PC) from 21 samples of AA broiler and LS chicken. (**a** and **b**) Total coefficient of variation of the top 4 PC in AA broiler; (**c** and **d**) total coefficient of variation of the top 4 PC in LS chicken.

**Additional file 8: Figure S3.** Comparison of the difference in developmental axis of physiological stages between two breeds. (**a** and **b**) The developmental axis from principal component (PC) 3 and PC 4. The red curve represents the developmental axis of AA broiler, and the blue curve represents the developmental axis of LS chicken. The X-axis represents the seven developmental stages, and the Y-axis represents the eigenvector. (**c** and **d**) Example of top and bottom 10% contributed transcripts through the developmental axis from PC 1 in AA broiler and LS chicken, respectively. The genes contained in the right gray box are the top 10% contributed transcripts for PC 1, and the genes contained in the left gray box are the bottom 10% contributed transcripts for PC 1. (**e**) GO enrichment analysis of the top 10% contributed transcripts from top 4 developmental axis in AA broiler and LS chicken, respectively. Bubble color indicates qvalue; size indicates gene numbers of the DET in GO terms. (**f**) The Venn plots show that the differences of the top 10% contributed transcripts come from the top 3 PC of AA broiler and top 4 PC of LS chicken.

**Additional file 9: Table S6.** PCA result. Each transcript’s eigenvector from the top 5 PC in AA broiler and LS chicken, respectively, and the transcript with the top 10% eigenvector in each PC between the two breeds. Furthermore, the top 10% contributed transcripts of the top PC involving myogenesis were found in  the top 3 PC of AA broiler, but did not exist in the top 4 PC of LS chicken.

**Additional file 10: Figure S4.** DET that affect myofiber development of breast muscle. The Venn plots show the DET of AA broiler vs. LS chicken comparison during (**a**) embryo and (**b**) hatched stages. Representative GO terms in the DET of AA broiler vs. LS chicken comparison during (**c**) embryo and (**d**) hatched stages. Bubble color indicates *q*-value; size indicates gene numbers of the DEG in GO terms.

**Additional file 11: Table S7.** Differential expressed transcripts between the two chicken breeds for each corresponding developmental stage.

**Additional file 12: Table S8.** Integration of the PCA results and differential expressed transcripts. Fifty-nine transcripts from 58 genes were significantly up-regulated in the breast muscle of AA broiler compared to LS chicken, including 44 genes that formed a molecular network responsible for muscle development and protein synthesis.

**Additional file 13: Table S9.** Alternative splicing events for 42 samples. List of all multi-isoform genes from this transcriptome, AS types of multi-isoform genes, and the distribution of different types of isoforms for each stage between the two breeds.

**Additional file 14: Figure S5.** Dynamic alternative splicing events during muscle development between the two chicken breeds. (**a**) Distribution of transcripts in chicken breast muscle at different developmental stages. Red dots indicate transcripts that are unique to each period, and dark red bars indicate transcripts common to all periods. (**b**) Distribution of different types of AS events. (**c**) Distribution of different types of AS events for each stage.

**Additional file 15: Figure S6.** Characteristics of the dynamic dominant transcripts during muscle development in chicken. (**a**) Number of transcripts exhibiting different folds of any other transcript within a given gene. (**b**) qPCR detection of the expression of the two isoforms at D1 in AA and LS chickens.

**Additional file 16: Table S10.** Identified dominant transcripts for each developmental stage of AA broiler and LS chicken, respectively. “Yes” indicates that this transcript is the dominant transcript in this sample, “No” indicates that this transcript is not the dominant transcript in this sample. Each period has three biological replicates that are named P1, P2 and P3.

**Additional file 17: Table S11.** Differentiated dominant transcripts between the two breeds. The multi-isoform genes exhibited switched dominant transcripts between AA broiler and LS chicken during muscle development.

**Additional file 18: Figure S7.** The switch of dominant transcripts of *TNFRSF6B* affects skeletal muscle development. (**a**) Genetic structure diagram of two *TNFRSF6B* isoforms. The black boxes represent coding sequences, and the grey boxes represent UTR sequences. The black line represent intron sequences. The region indicated by the red solid line represents the fragment amplified by qPCR. The primers for TNFRSF6B-X2 can just amplify 126 bp of exon 1 (5'UTR). The primers for TNFRSF6B-X1 can just amplify 139 bp of exon 1 (5'UTR and a small part of the coding sequence). The region indicated by the green dotted line is the coding sequence (135 bp) unique to *TNFRSF6B*-X1. (**b**) Motif analysis of two isoforms of *TNFRSF6B*. **c** RT-qPCR detected the expression of two isoforms at D1 in AA broiler and LS chicken, respectively. The *p*-value in **b** is defined as the probability that a random sequence (with the same length and conforming to the background) would have a match to the motif under test with a score greater or equal to the largest value found in the sequence under test.

## Data Availability

All data generated or analysed during this study are included in this published article and its Additional files. The RNA-seq raw data of 42 RNA libraries were deposited in NCBI Sequence Read Archive (NCBI-SRA) with accession number PRJNA729271.
