# Supplementary material for: Comparative analyses of dynamic transcriptome profiles highlight key response genes and dominant isoforms for muscle development and growth in chicken
Source: Genet Sel Evol. 2023 Oct 23;55:73. doi: 10.1186/s12711-023-00849-4 (PMC10591418; doi:10.1186/s12711-023-00849-4)
Supplement: Supplementary file 2 — Additional file 2: Table S2. Summary of sequencing quality: raw reads, clean reads, mapping rate, Q20 and Q30 in this study. [file 12711_2023_849_MOESM2_ESM.doc]

**Table S1** Summary of sequencing quality in this study

| **Sample name** | **Raw reads** | **Clean reads** | **clean bases** | **Total mapped** | **Error rate**  **(%)** | **Q20**  **(%)** | **Q30**  **(%)** | **GC content**  **(%)** |
| --- | --- | --- | --- | --- | --- | --- | --- | --- |
| AA1D_P1 | 89124602 | 87225524 | 13.08G | 94.43% | 0.01 | 97.35 | 93.04 | 50.36 |
| AA1D_P2 | 90632434 | 87096270 | 13.06G | 91.15% | 0.02 | 94.47 | 86.73 | 57.07 |
| AA1D_P3 | 106799514 | 103395392 | 15.51G | 92.31% | 0.02 | 97.08 | 92.63 | 52.36 |
| AA1W_P1 | 81806862 | 79721570 | 11.96G | 90.69% | 0.02 | 97.09 | 92.41 | 55.08 |
| AA1W_P2 | 102632394 | 99809308 | 14.97G | 90.58% | 0.02 | 97.1 | 92.42 | 56.25 |
| AA1W_P3 | 101312358 | 99355986 | 14.9G | 90.50% | 0.02 | 97.07 | 92.37 | 55.27 |
| AA3W_P1 | 113662528 | 106949350 | 16.04G | 91.86% | 0.02 | 96.41 | 91.08 | 55.89 |
| AA3W_P2 | 97313048 | 92764186 | 13.91G | 92.09% | 0.02 | 96.46 | 91.19 | 55.46 |
| AA3W_P3 | 105095350 | 99725084 | 14.96G | 91.77% | 0.02 | 96.45 | 91.17 | 56.06 |
| AA5W_P1 | 93914792 | 90479648 | 13.57G | 88.43% | 0.03 | 93.89 | 85.73 | 55.48 |
| AA5W_P2 | 108998038 | 104725984 | 15.71G | 85.52% | 0.03 | 92.95 | 85.8 | 54.46 |
| AA5W_P3 | 84294658 | 80737860 | 12.11G | 89.88% | 0.03 | 93.95 | 85.91 | 57.19 |
| AAE10P1 | 93079562 | 89223638 | 13.38G | 90.95% | 0.02 | 94.56 | 87.33 | 50.24 |
| AAE10P2 | 106977070 | 102613692 | 15.39G | 91.11% | 0.02 | 94.65 | 87.52 | 51.05 |
| AAE10P3 | 94524558 | 89906364 | 13.49G | 91.81% | 0.02 | 94.95 | 88.31 | 52.49 |
| AAE14P1 | 85508224 | 82340970 | 12.35G | 89.93% | 0.03 | 93.99 | 85.95 | 54.12 |
| AAE14P2 | 90910094 | 88070898 | 13.21G | 89.60% | 0.03 | 93.77 | 85.48 | 54.48 |
| AAE14P3 | 106109444 | 101829894 | 15.27G | 92.63% | 0.02 | 94.88 | 87.58 | 55.32 |
| AAE18P1 | 87844576 | 82132676 | 12.32G | 93.85% | 0.03 | 94.27 | 86.37 | 56.35 |
| AAE18P2 | 99318238 | 92894764 | 13.93G | 94.02% | 0.03 | 94.11 | 86.04 | 58.07 |
| AAE18P3 | 91685302 | 87918984 | 13.19G | 92.91% | 0.02 | 94.87 | 87.51 | 56.25 |
| LS1D_P1 | 106263764 | 102169706 | 15.33G | 86% | 0.03 | 93.76 | 85.45 | 52.4 |
| LS1D_P2 | 117541708 | 109292470 | 16.39G | 93.52% | 0.02 | 97 | 92.51 | 53.64 |
| LS1D_P3 | 101844544 | 97033112 | 14.55G | 88.82% | 0.03 | 93.46 | 85.01 | 56.99 |
| LS1W_P1 | 101288278 | 98694992 | 14.8G | 89.96% | 0.02 | 97.05 | 92.36 | 54.15 |
| LS1W_P2 | 105444804 | 102852440 | 15.43G | 88.60% | 0.02 | 96.59 | 91.31 | 55.23 |
| LS1W_P3 | 92005966 | 89789812 | 13.47G | 90.35% | 0.02 | 96.82 | 91.78 | 54.26 |
| LS3W_P1 | 92200670 | 90086900 | 13.51G | 90.28% | 0.02 | 96.76 | 91.67 | 55.73 |
| LS3W_P2 | 82780390 | 80688388 | 12.1G | 91.32% | 0.02 | 96.65 | 91.46 | 51.51 |
| LS3W_P3 | 86298314 | 83778568 | 12.57G | 90.90% | 0.02 | 96.69 | 91.52 | 51.3 |
| LS5W_P1 | 86039090 | 84115182 | 12.62G | 92.12% | 0.02 | 97.19 | 92.59 | 55.5 |
| LS5W_P2 | 106029536 | 101525490 | 15.23G | 84.92% | 0.03 | 92.93 | 85.87 | 54.72 |
| LS5W_P3 | 88569064 | 86571172 | 12.99G | 92.78% | 0.02 | 97.28 | 92.79 | 56.29 |
| LSE10P1 | 96216604 | 92398740 | 13.86G | 90.39% | 0.02 | 94.74 | 87.7 | 49.52 |
| LSE10P2 | 92755974 | 88661648 | 13.3G | 91.43% | 0.02 | 94.93 | 88.29 | 51.59 |
| LSE10P3 | 93430936 | 89502662 | 13.43G | 89.70% | 0.02 | 94.22 | 86.66 | 51.11 |
| LSE14P1 | 89816634 | 86283306 | 12.94G | 89.47% | 0.03 | 93.84 | 85.65 | 51.49 |
| LSE14P2 | 101090242 | 95704908 | 14.36G | 89.48% | 0.03 | 93.91 | 85.98 | 53.96 |
| LSE14P3 | 92714800 | 86912434 | 13.04G | 93.37% | 0.03 | 94.36 | 86.55 | 55.36 |
| LSE18P1 | 89797586 | 86369390 | 12.96G | 92.06% | 0.02 | 94.72 | 87.2 | 57.41 |
| LSE18P2 | 85289970 | 82166808 | 12.33G | 92.41% | 0.02 | 94.97 | 87.78 | 55.65 |
| LSE18P3 | 95461784 | 91688958 | 13.75G | 92.78% | 0.02 | 94.8 | 87.39 | 56.57 |
