# Supplementary figures and images for: Comparative analyses of dynamic transcriptome profiles highlight key response genes and dominant isoforms for muscle development and growth in chicken
Source: Genet Sel Evol. 2023 Oct 23;55:73. doi: 10.1186/s12711-023-00849-4 (PMC10591418; doi:10.1186/s12711-023-00849-4)

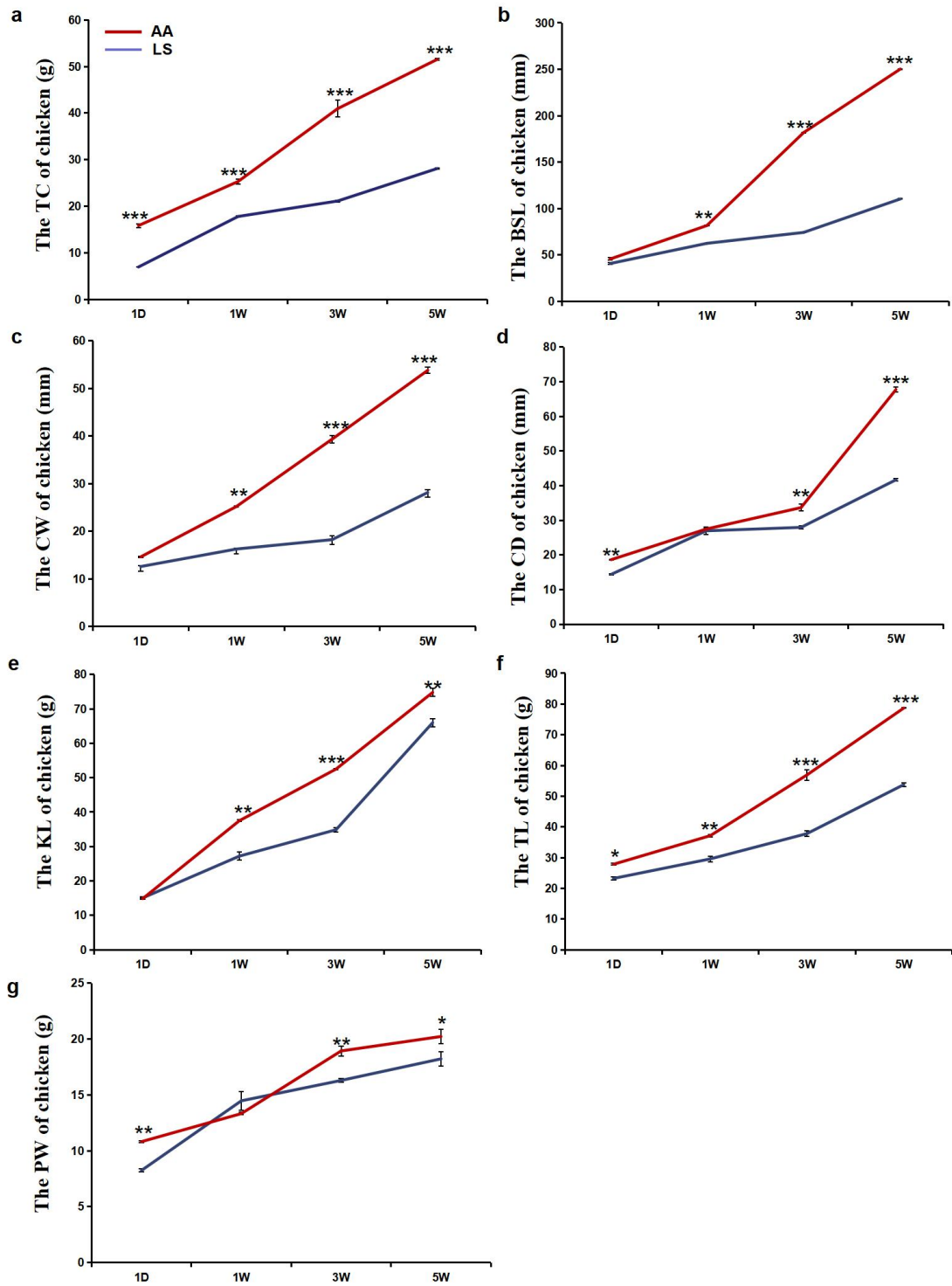

Supplement: Supplementary file 3 — Additional file 3: Figure S1. Comparison of body size phenotypes: (a) TC: tibial circumference; (b) BSL: body slanting length; (c) CW: breast width; (d) CD: breast depth; (e) KL: Keel length; (f) PW: pelvis width; (g) TL: tibial length. [file 12711_2023_849_MOESM3_ESM.pdf]

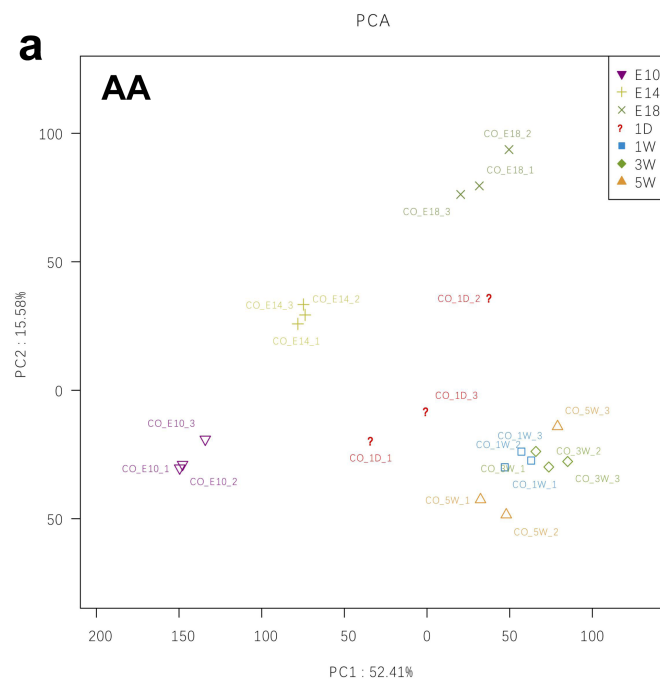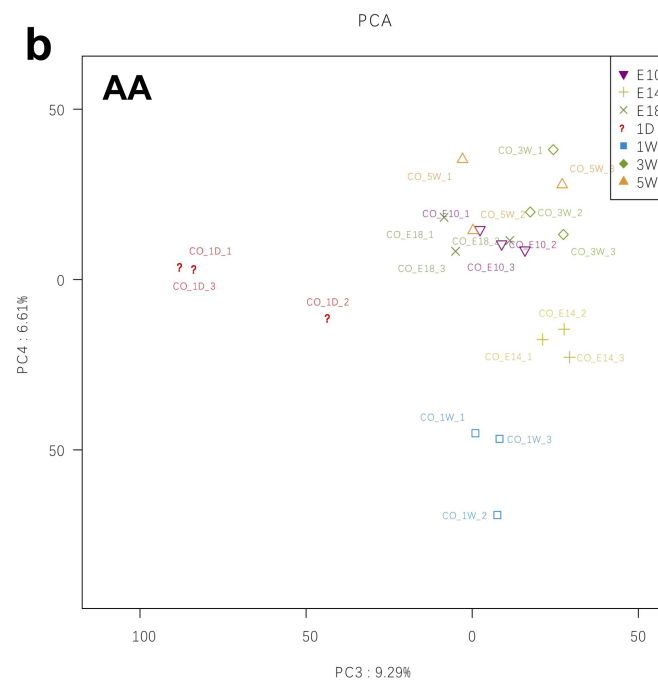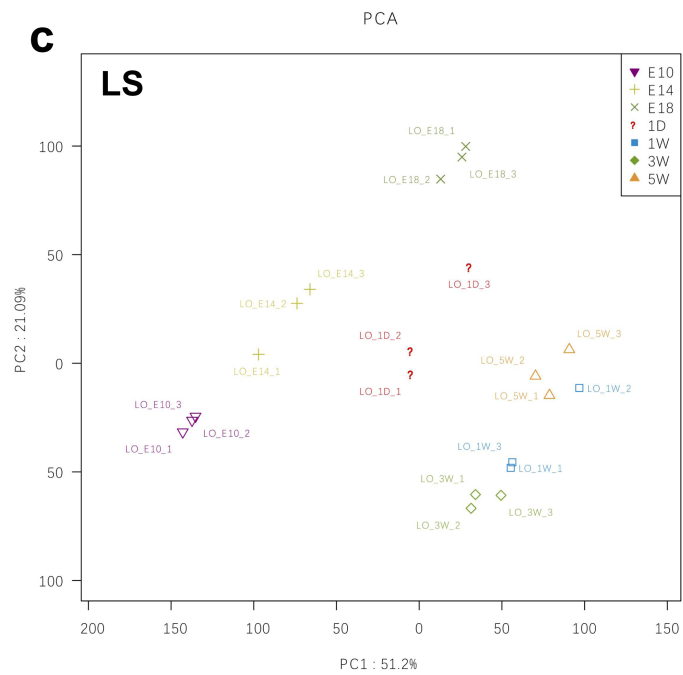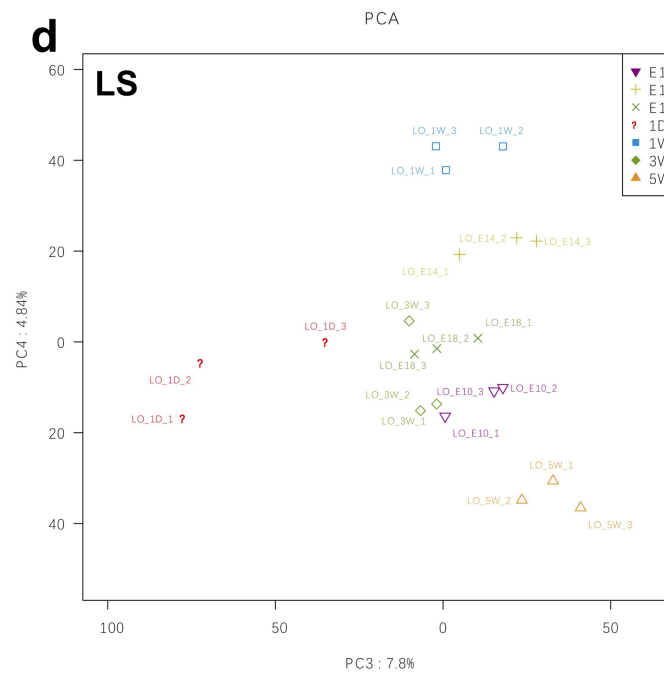

Supplement: Supplementary file 7 — Additional file 7: Figure S2. Top 4 principal components (PC) from 21 samples of AA broiler and LS chicken. (a and b) Total coefficient of variation of the top 4 PC in AA broiler; (c and d) total coefficient of variation of the top 4 PC in LS chicken. [file 12711_2023_849_MOESM7_ESM.pdf]

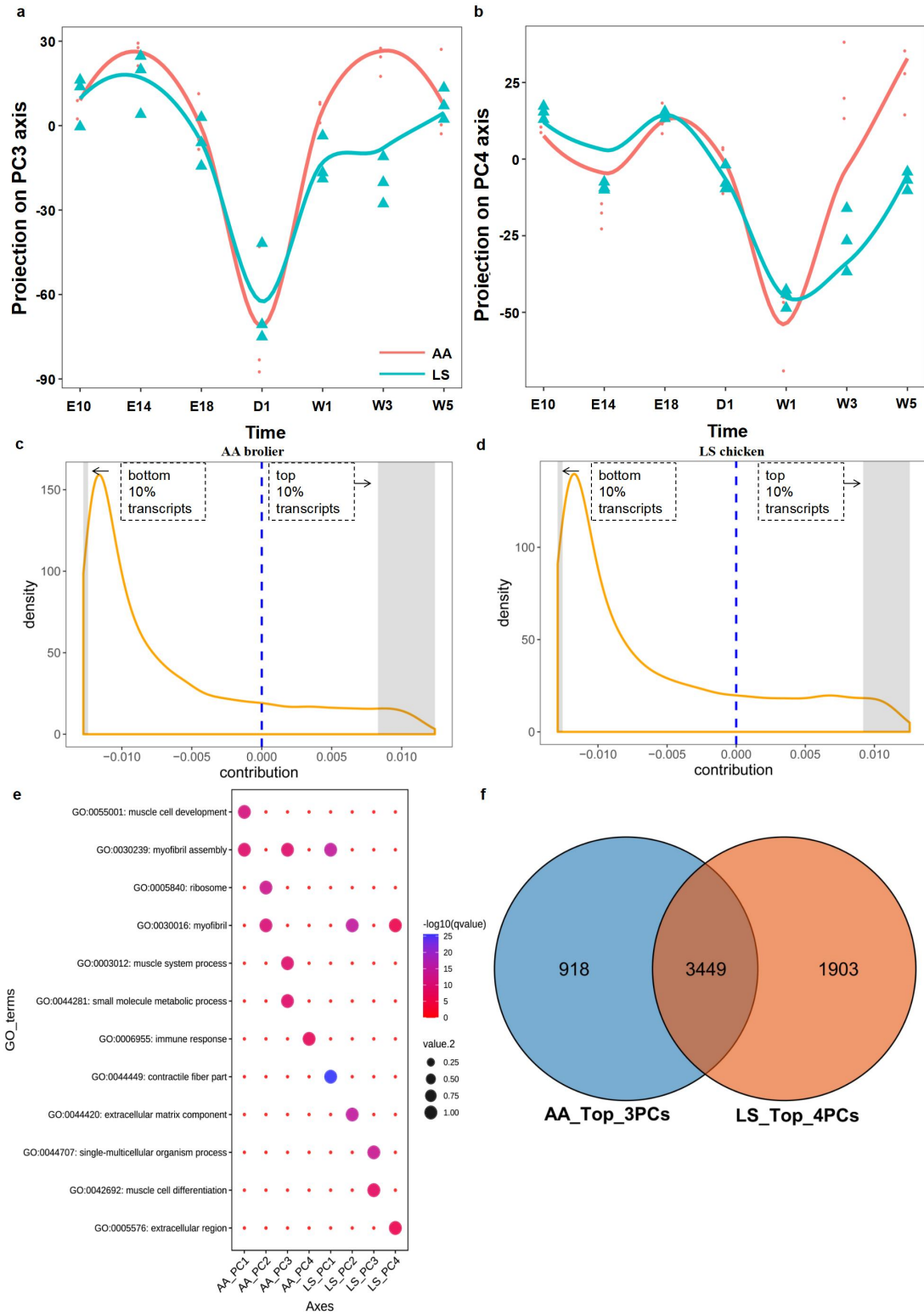

Supplement: Supplementary file 8 — Additional file 8: Figure S3. Comparison of the difference in developmental axis of physiological stages between two breeds. (a and b) The developmental axis from principal component (PC) 3 and PC 4. The red curve represents the developmental axis of AA broiler, and the blue curve represents the developmental axis of LS chicken. The X-axis represents the seven developmental stages, and the Y-axis represents the eigenvector. (c and d) Example of top and bottom 10% contributed transcripts through the developmental axis from PC 1 in AA broiler and LS chicken, respectively. The genes contained in the right gray box are the top 10% contributed transcripts for PC 1, and the genes contained in the left gray box are the bottom 10% contributed transcripts for PC 1. (e) GO enrichment analysis of the top 10% contributed transcripts from top 4 developmental axis in AA broiler and LS chicken, respectively. Bubble color indicates qvalue; size indicates gene numbers of the DET in GO terms. (f) The Venn plots show that the differences of the top 10% contributed transcripts come from the top 3 PC of AA broiler and top 4 PC of LS chicken. [file 12711_2023_849_MOESM8_ESM.pdf]

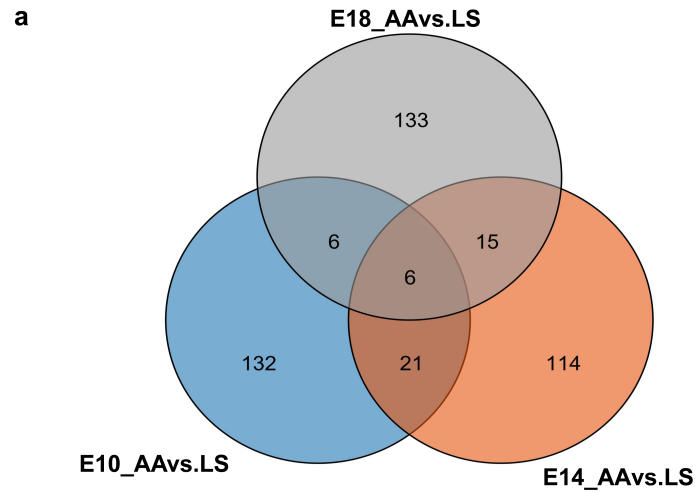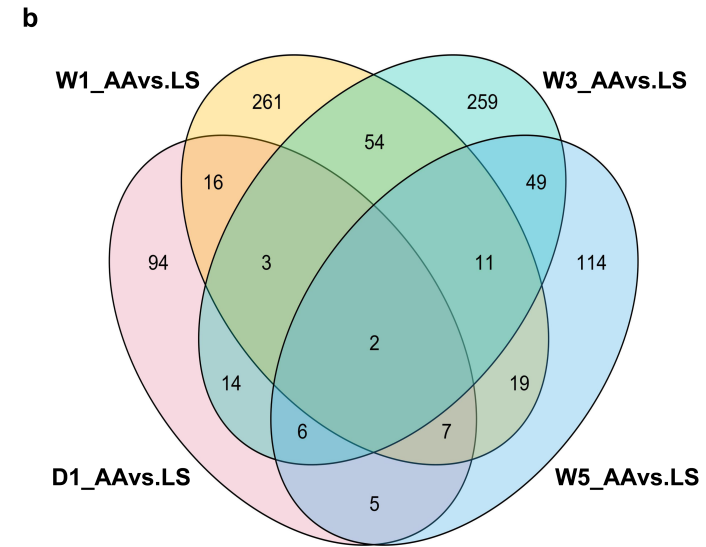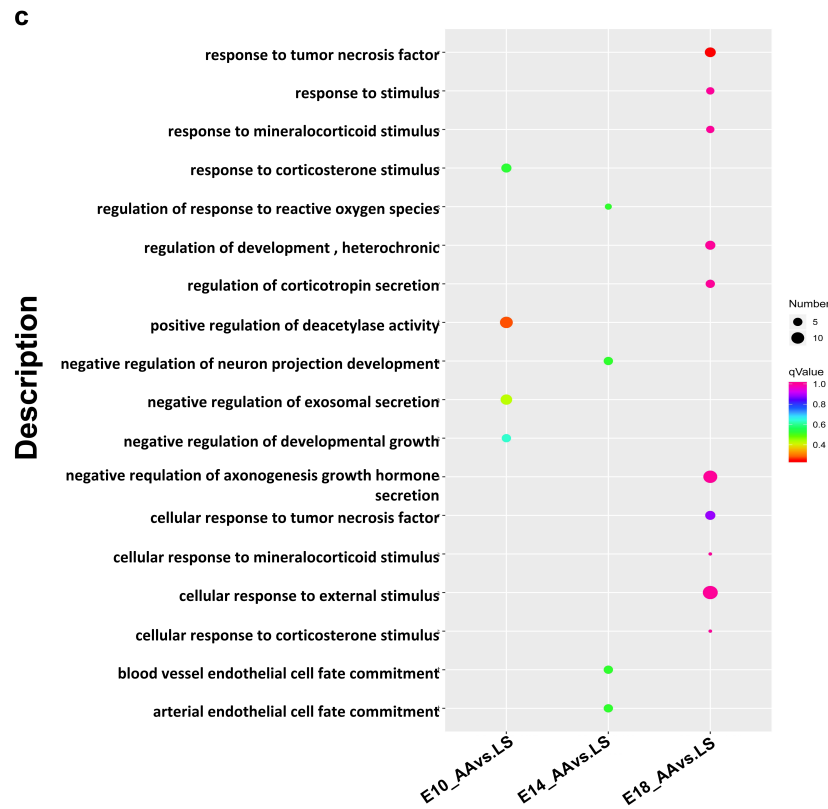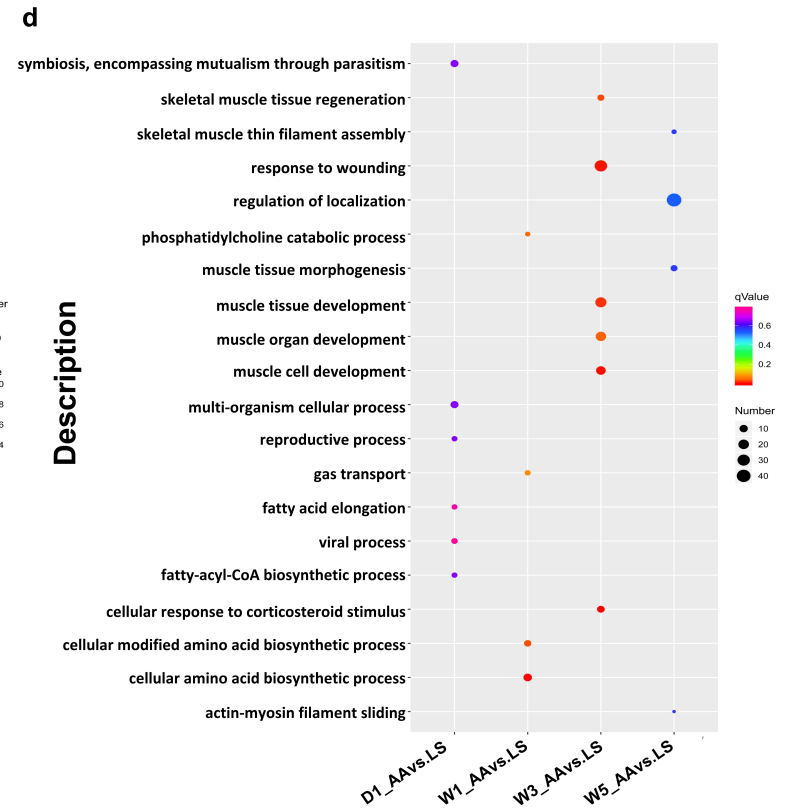

Supplement: Supplementary file 10 — Additional file 10: Figure S4. DET that affect myofiber development of breast muscle. The Venn plots show the DET of AA broiler vs. LS chicken comparison during (a) embryo and (b) hatched stages. Representative GO terms in the DET of AA broiler vs. LS chicken comparison during (c) embryo and (d) hatched stages. Bubble color indicates q-value; size indicates gene numbers of the DEG in GO terms. [file 12711_2023_849_MOESM10_ESM.pdf]

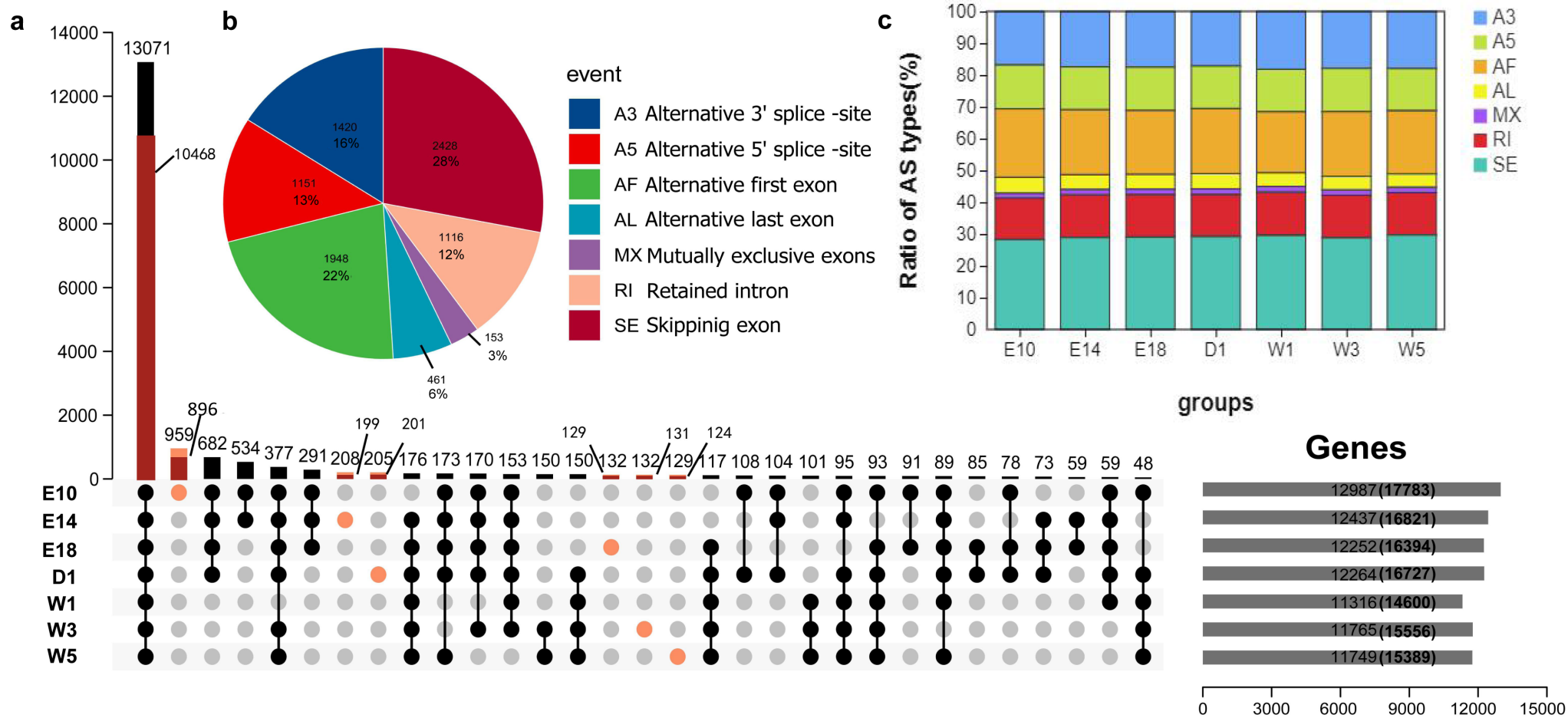

Supplement: Supplementary file 14 — Additional file 14: Figure S5. Dynamic alternative splicing events during muscle development between the two chicken breeds. (a) Distribution of transcripts in chicken breast muscle at different developmental stages. Red dots indicate transcripts that are unique to each period, and dark red bars indicate transcripts common to all periods. (b) Distribution of different types of AS events. (c) Distribution of different types of AS events for each stage. [file 12711_2023_849_MOESM14_ESM.pdf]

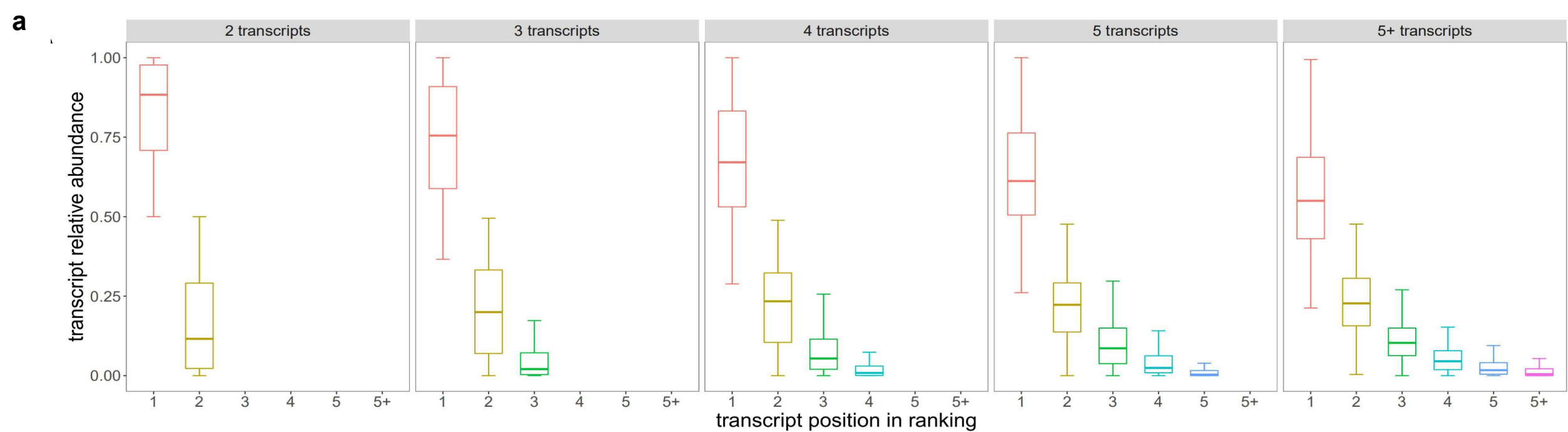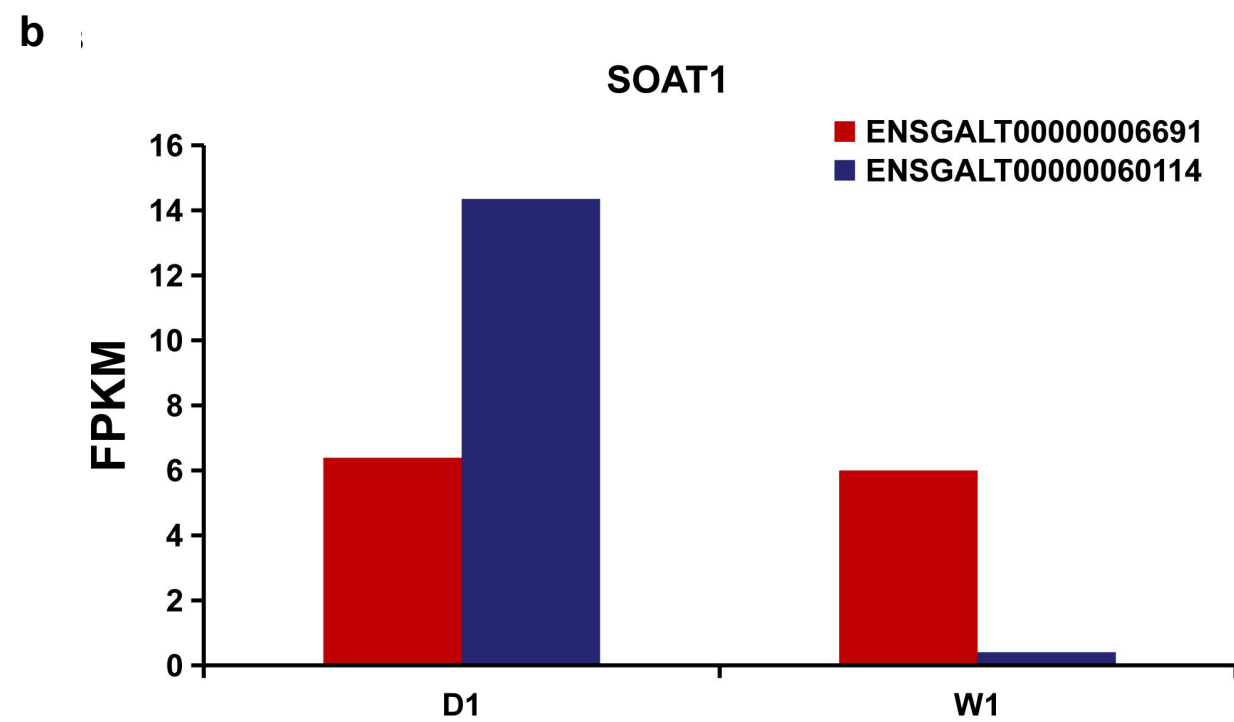

Supplement: Supplementary file 15 — Additional file 15: Figure S6. Characteristics of the dynamic dominant transcripts during muscle development in chicken. (a) Number of transcripts exhibiting different folds of any other transcript within a given gene. (b) qPCR detection of the expression of the two isoforms at D1 in AA and LS chickens. [file 12711_2023_849_MOESM15_ESM.pdf]
