# Supplementary material for: Comparative analyses of dynamic transcriptome profiles highlight key response genes and dominant isoforms for muscle development and growth in chicken
Source: Genet Sel Evol. 2023 Oct 23;55:73. doi: 10.1186/s12711-023-00849-4 (PMC10591418; doi:10.1186/s12711-023-00849-4)
Supplement: Supplementary file 18 — Additional file 18: Figure S7. The switch of dominant transcripts of TNFRSF6B affects skeletal muscle development. (a) Genetic structure diagram of two TNFRSF6B isoforms. The black boxes represent coding sequences, and the grey boxes represent UTR sequences. The black line represent intron sequences. The region indicated by the red solid line represents the fragment amplified by qPCR. The primers for TNFRSF6B-X2 can just amplify 126 bp of exon 1 (5'UTR). The primers for TNFRSF6B-X1 can just amplify 139 bp of exon 1 (5'UTR and a small part of the coding sequence). The region indicated by the green dotted line is the coding sequence (135 bp) unique to TNFRSF6B-X1. (b) Motif analysis of two isoforms of TNFRSF6B. c RT-qPCR detected the expression of two isoforms at D1 in AA broiler and LS chicken, respectively. The p-value in b is defined as the probability that a random sequence (with the same length and conforming to the background) would have a match to the motif under test with a score greater or equal to the largest value found in the sequence under test. [file 12711_2023_849_MOESM18_ESM.pdf]

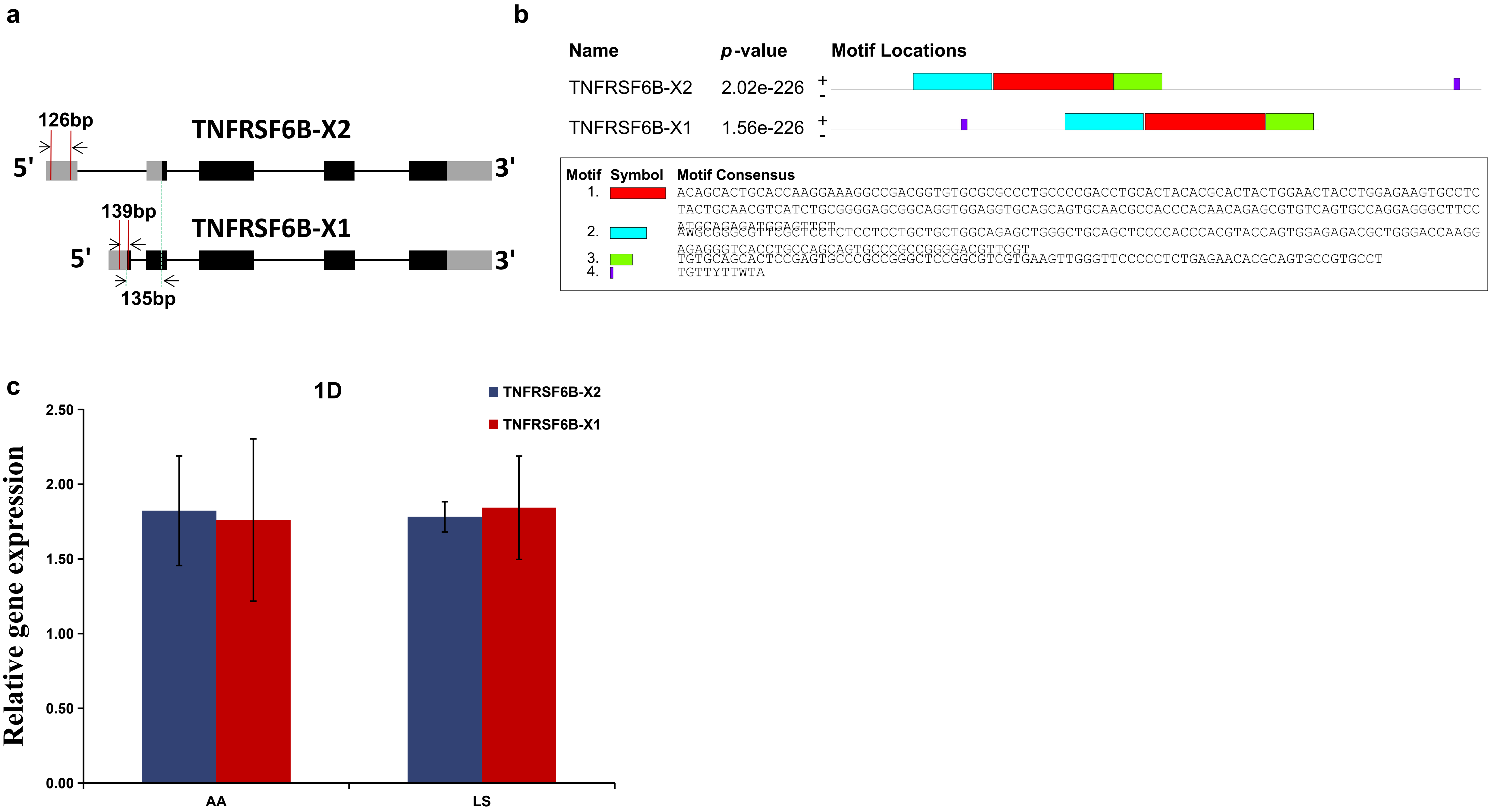

a
126bp
TNFRSF6B-X2
5'
3'
139bp
TNFRSF6B-X1
5'
3'
135bp
b
Name
p-value
Motif Locations
TNFRSF6B-X2
2.02e-226
TNFRSF6B-X1
1.56e-226
+
-
Motif
Symbol
Motif Consensus
1.
ACAGCACTGCACCAAGGAAAGGCCGACGGTGTGCGCGCCCTGCCCCGACCTGCACTACACGCACTACTGGAACTACCTGGAGAAGTGCCTC
TACTGCAACGTCATCTGCGGGGAGCGGCAGGTGGAGGTGCAGCAGTGCAACGCCACCCACAACAGAGCGTGTCAGTGCCAGGAGGGCTTCC
ATGCAGAGATGGAGTTCT
2.
AWGCGGGCGTTCGCTCCTCTCCTCCTGCTGCTGGCAGAGCTGGGCTGCAGCTCCCCACCCACGTACCAGTGGAGAGACGCTGGGACCAAGG
AGAGGGTCACCTGCCAGCAGTGCCCGCCGGGGACGTTCGT
3.
TGTGCAGCACTCCGAGTGCCCGCCGGGCTCCGGCGTCGTGAAGTTGGGTTCCCCCTCTGAGAACACGCAGTGCCGTGCCT
4.
TGTTYTTWTA
c
1D
TNFRSF6B-X2
TNFRSF6B-X1
Relative gene expression
2.50
2.00
1.50
1.00
0.50
0.00
AA
LS
